# Hypoxia-inducible factor 2α does not regulate cortical brain development

**DOI:** 10.1371/journal.pone.0359507

**Published:** 2026-10-14

**Authors:** Jennifer Lanto, Laura Rohmann, Tim Theege, Valeriia Abramenko, Lisa Große, Andreas Madlener, Julia Anna Elisabeth Neitzel, Alexander Storch, Franz Markert

**Affiliations:** 1 Department of Neurology, University of Rostock, Rostock, Mecklenburg-Western Pomerania, Germany; 2 German Centre for Neurodegenerative Diseases (DZNE) Rostock-Greifswald, Rostock, Mecklenburg-Western Pomerania, Germany; Keio University School of Medicine Graduate School of Medicine: Keio Gijuku Daigaku Igakubu Daigakuin Igaku Kenkyuka, JAPAN

## Abstract

Dynamic changes in physiological oxygen levels are crucial for proper cortical brain development. In this context, oxygen is important not only for its role in energy metabolism but also as a signaling molecule. While much is already known about its most prominent signaling factor hypoxia-inducible factor (HIF)-1α, little is known about its functionally similar isoform HIF-2α during cortical development. Therefore, we utilized HIF-2α conditional knock-out mice (Emx1 and Nestin driven), which were kept under maternal hypoxia (10% pO_2_), physioxia (21% pO_2_), or hyperoxia (75% pO_2_) starting at E14.5 for 48 h and analyzed their cortical development by immunohistochemistry at E16.5. The analysis of newborn neurons (BrdU^+^) and cortical layer IV (Tbr1^+^), V (Ctip2^+^), and upper layer neurons (Satb2^+^) revealed clear effects of oxygen, with hyperoxia particularly increasing the number of Ctip2^+^/Tbr1^-^ layer V neurons in E16.5 fetuses. However, neither the number of newborn neurons nor the number of layer-specific neurons was affected by HIF-2α CKO. Similarly, oxygen-related effects on neural stem cells (NSCs) and neuroprogenitor cells were not mediated by HIF-2α in E16.5 fetuses. Contributing, *in vitro* expansion and stem cell maintenance of NSCs were accelerated by physioxia (3% pO_2_) compared to hyperoxia (21% pO_2_), without involvement of HIF-2α under either condition. In conclusion, oxygen modulates cortical development, but HIF-2α does not mediate these effects. Thus, oxygen-related changes in neurogenesis and cortical morphology appear to be regulated via HIF-2α-independent mechanisms.

## Introduction

Oxygen is crucial during brain development, and altered levels of oxygen already lead to severe changes in cortical development [1–3]. Besides its essential function regarding energy metabolism, oxygen additionally acts as an important signaling factor via hypoxia-inducible factors (HIFs) [4–7]. HIFs are transcription factors that are permanently expressed, but their α-subunits are rapidly hydroxylated by oxygen-dependent proline hydroxylases and subsequently degraded by von Hippel-Lindau tumor suppressor protein under higher oxygen conditions [8–11]. Under lower oxygen conditions, the hydroxylation of HIFs fails, and they are able to enter the nucleus where they bind to the HIF-β-subunit and act as transcription factor for genes with a hypoxia response element [12]. While there is only one HIF-β-subunit known, there are three α-subunits named HIF-1α, HIF-2α, and HIF-3α. All α-subunits contain an oxygen-dependent degradation domain, however, only HIF-1α and HIF-2α have a C-terminal transactivation domain, which is necessary for transcription factor activity [13].

*In vitro* experiments have shown that the transcriptional activity of HIF-1α can influence major properties of neural stem and progenitor cells, such as proliferation and stem cell maintenance [14–17]. Although these studies were able to show an influence of oxygen on various genes mediated by HIF-1α, there are still inconsistencies that suggest that other factors, such as HIF-2α, could play a crucial role [18,19]. Both HIF-1α and HIF-2α can activate genes via their hypoxia response element [20]. However, both HIFs differentially regulate gene expression, with HIF-2α activating a much broader range of genes [21]. Thereby, HIF-2α, but not HIF-1α or HIF-3α, can regulate cell proliferation in human embryonic stem cells, indicating an important role in stem cell proliferation [22].

During cortical brain development, altered oxygen levels have been proven to affect neurogenesis [2,23–25], however, the involvement of HIFs remains in part enigmatic. A Nestin-driven conditional knock-out of HIF-1α starting at around E12.5 resulted in a severe reduction of angiogenesis but only minor effects regarding stem cell proliferation at E16 [23,26]. An Emx1-driven knockdown model of HIF-1α, in which Cre activity and subsequently the conditional knock-out occur earlier at around E10.5 and more efficiently, led to an expansion of the stem and progenitor cell pool and a reduction of cortical thickness at E13.5 [2,27]. The same group could not detect HIF-2α protein in E13.5 Gpr124^KO^ mouse brains, a model with brain-specific impairment of angiogenesis or controls [2]. Nevertheless, Kleszka and colleagues demonstrated that a Nestin-Cre driven HIF-2α conditional knock-out led to a loss of cortical width and pyramidal cells as well as altered cognitive functions in young adult (P70) mice [28]. Of note, Kleszka and co-workers assessed the effects of the Nestin-driven HIF-2α CKO effectuated in the early to mid-neurogenesis phase at around E13.5 only upon substantial completion of brain development in young adulthood at P70 [28]. Timing and underlying mechanisms of this phenomenon remain unclear and may not necessarily be attributed to impaired neurogenesis during fetal cortical brain development. However, the authors also showed reduced migration and cell viability in primary HIF-2α CKO neural stem cells (NSCs), suggesting a critical role for HIF-2α in these cells [28].

Together, it remains enigmatic how HIF-2α affects NSCs during the dynamic process of cortical brain development *in vivo*. The fact that NSC production for generating upper layer neurons – where most of the pyramidal neurons are located – peaks between E14.5 and E16.5 [29] in conjunction with the known impact of HIF-2α on stem cell proliferation under *in vitro* conditions, prompted us to investigate the role of HIF-2α on early to mid-neurogenesis. We used the already discussed Nestin-Cre driven (starting at E12.5/E13.5) HIF-2α CKO [27,30] and, in addition, an Emx1-driven HIF-2α CKO effectuated at E10.5, which covers Cre expression more sufficiently at earlier stages, and investigated cortical brain development in E16.5 fetuses.

## Materials and methods

### Animals

All animals were maintained and treated with permission from the local Department of Animal Welfare (Ministerium für Klimaschutz, Landwirtschaft, Ländliche Räume und Umwelt Mecklenburg-Vorpommern, Referat VI 500 – Tierschutz, reference numbers 7221.3-1-043/16 and 7221.3-1-012/21) and complied with the Tierschutzgesetz and Verordnung zur Umsetzung der Richtlinie 2010/63/EU from Germany as well as ARRIVE guidelines. In addition to the predefined experimental endpoints, the study included several humane endpoints to minimize unnecessary burden on the animals. These included reduced food intake, uncoordinated movements, severe weight loss, pronounced respiratory distress, and miscarriages. Animal health and behavior were monitored and scored daily, including assessments of body weight, spontaneous behavior, normal movement, and the condition of fur, body orifices, eyes, and face. A predefined combined score indicative of severe burden was additionally used as a humane endpoint. No animal reached a humane endpoint or died before completion of the experiments. The number of animals used is indicated for each experiment. The total duration of each experiment prior to brain dissection for morphological analyses was 48 h. For neural stem cell isolation and DNA/RNA extraction from *in vivo* samples, no prior treatment was performed. No surgical procedures or other painful interventions were performed during the study, and therefore no anesthesia or analgesia was required during the experimental procedures. Animals were euthanized by cervical dislocation without prior anesthesia to minimize the interval between euthanasia and tissue collection and thereby enable rapid processing of the brain tissue. To alleviate suffering and promote animal welfare, the animals were housed in standardized, enriched cages under a 12 h light/dark cycle and had *ad libitum* access to food and water both prior to and during the experimental period. Further, all animals were handled by the same experimenter before the start of the experiments for habituation purposes. To increase the accuracy of pregnancy detection, mice were checked for the presence of a vaginal plug after overnight mating and monitored for weight gain afterwards. All researchers involved in the animal experiments were trained in the applied methods, received continuous education in animal experimentation, and were authorized to perform the procedures by the local Department of Animal Welfare.

To generate HIF-2α-CKO mice, Epas1^tm1Mcs^/J (HIF-2α^flox/flox^; Stock: 008407; RRID: IMSR_JAX:008407) mice were crossed with either B6.Cg-Tg(Nes-Cre)1Kln/J (NesCre-HIF-2α-CKO; Stock: 003771; RRID: IMSR_JAX:003771) or B6.129S2-Emx1^tm1(Cre)Krj^/J (Emx1-HIF-2α-CKO; Stock: 005628; RRID: IMSR_JAX:005628), all purchased from The Jackson Laboratory (Bar Harbor, USA). The resulting HIF-2α CKO male mice were mated with HIF-2α^flox/flox^ female mice for one night, and pregnant mice were subsequently used for the experiments. Neither the morphology of the embryos nor the reproductive numbers of the individual litters showed any detectable changes as a result of the genetic knock-out. After successful mating, BrdU (25 mg/kg body weight) (SERVA Electrophoresis, Heidelberg, Germany) was injected for birth-dating analyses of newly generated neurons at E14.5 for analysis of E16.5 fetuses. Birth-dating datasets were obtained for NesCre-HIF-2α CKO mice, whereas for unknown reasons, no reliable data could be obtained for the Emx1-HIF-2α-CKO line. Following BrdU administration, pregnant mice were placed in a glovebox (InerTec, Grenchen, Switzerland) with 10%, 21% or 75% pO2 for 48 hours (Fig 1a).

**Fig 1:**
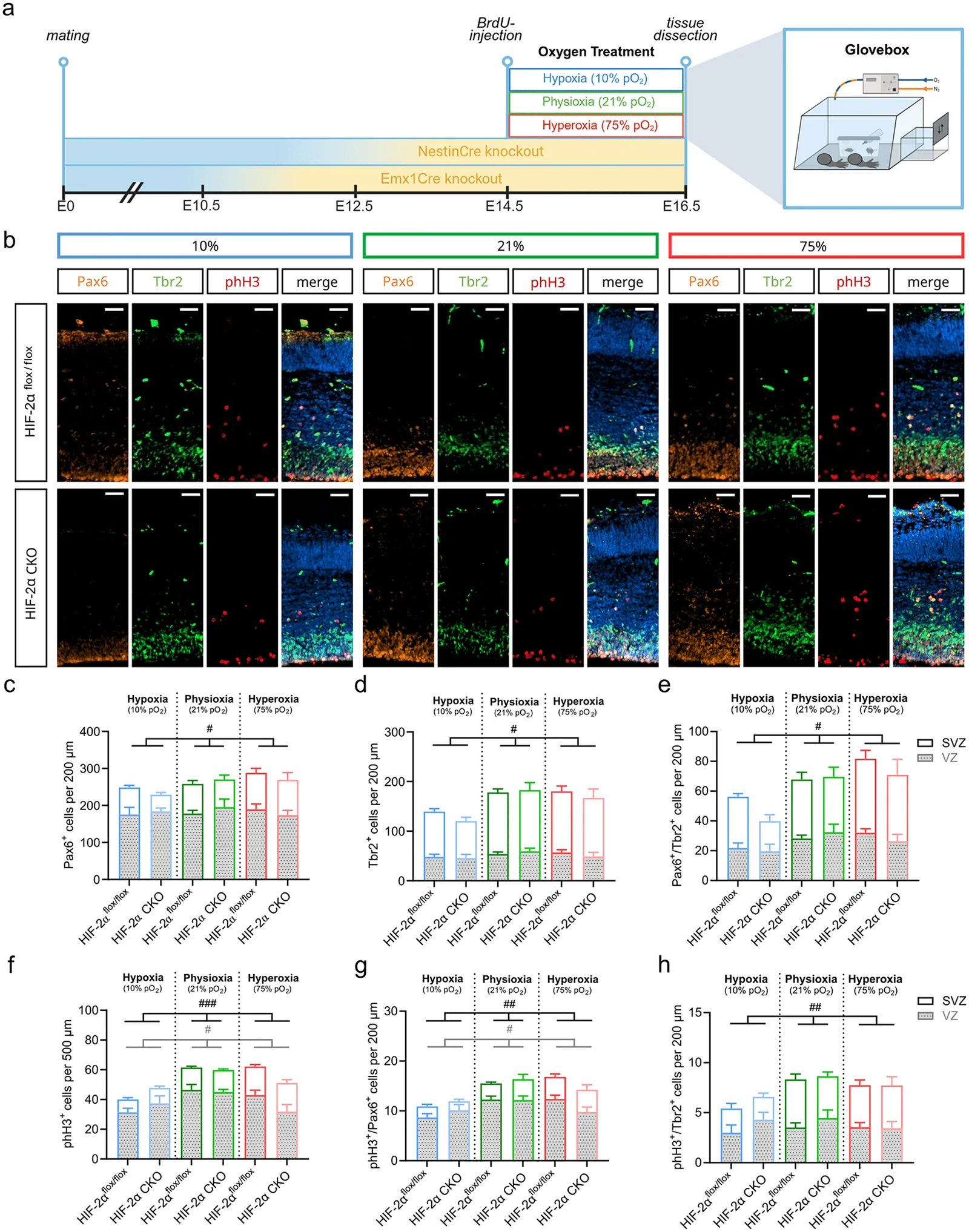
Effects of Emx1-HIF-2α CKO on neural stem/progenitor cell composition and proliferation in the developing mouse cortex at E16.5. (a) Graphical representation of the experimental design. After mice were mated at a defined time-point, pregnant mice received a BrdU injection on E14.5, immediately before maternal oxygen treatment for 48 h with 10% pO_2_, 21% pO_2,_ or 75% pO_2_. Tissue dissection was performed on E16.5. NesCre-HIF-2α CKO extends from ~E12.5 onwards, and Emx1-driven CKO starts earlier at ~E10.5. (b) Representative images of Pax6^+^ neural stem cells (orange), Tbr2^+^ intermediate progenitor cells (green) and phH3^+^ mitotic cells (red) in the developing cortex of E16.5 HIF-2α^flox/flox^ mice (top panels) and Emx1-HIF-2α CKO mice (bottom panels) kept under hypoxia (10% pO_2_, left panel), physioxia (21% pO_2_, middle panel) or hyperoxia (75% pO_2_, right panel) for 48 h. Cell nuclei were counterstained with Hoechst (blue). Scale bar = 50 μm. (c-f) Quantification of Pax6 ⁺ cells (c), Tbr2 ⁺ cells (d), Pax6 ⁺ /Tbr2 ⁺ cells (e), phH3^+^/Pax6^+^ (g), phH3^+^/Tbr2^+^ (h) cells per 200 μm and phH3 ⁺ cells (f) per 500 μm cortical column in E16.5 HIF-2α^flox/flox^ and Emx1-HIF-2α CKO mice kept under the three oxygen conditions. Cells were separately counted in the ventricular zone (VZ; gray with dotted fill) and subventricular zone (SVZ; white). Statistical analysis was performed by using two-way ANOVA or robust two-way ANOVA (c-h). # indicates significance in the main effect of oxygen (gray for VZ, black for SVZ), # p ≤ 0.05, ## p < 0.01, ### p < 0.001. Data are presented as mean ± s.e.m (n ≥ 4). *p*-values from *post-hoc* t-test with Bonferroni adjustment are omitted from the diagram for clarity. For full statistics for (c-h), see S1-S6 Tables in S1 File.

Following the exposure period, the animals were euthanized as described above, after which the embryos were immediately collected for further processing. The entire brain of each embryo was dissected, fixed in 4% PFA for 24 hours, dehydrated in 30% sucrose for at least 24 hours, and then snap-frozen. The brains were subsequently sectioned coronally at 20 µm thickness using a cryomicrotome (Leica CM3050, Leica Biosystems, Nussloch, Germany) and mounted on Superfrost Plus slides (Thermo Fisher Scientific, Waltham, USA), which were stored at 4°C until staining.

### Genotyping

To distinguish between HIF-2α^flox/flox^ and HIF-2α CKO fetuses, DNA was isolated from their tails. The tissue was incubated in lysis buffer (100 nM Tris-HCl, 5 mM EDTA, 0.2% SDS, 200 mM NaCl, 20 mg/ml proteinase K) at 56 °C overnight, followed by precipitation in isopropanol and a washing step with 70% ethanol. The DNA concentration was determined using a Nanodrop (Thermo Fisher Scientific), and a touch-down PCR was performed with the following primers (*HIF-2α*: forward primer: GAGAGCAGCTTCTCCTGGAA, reverse primer: TGTAGGCAAGGAAACCAAGG; Cre: forward primer: GCGGTCTGGCAGTAAAAACTATC, reverse primer: GTGAAACAGCATTGCTGTCACTT). After PCR, the samples were mixed with a DNA dye (EVAGREEN^®^/GelGreen, Merck, Darmstadt, Germany) and a DNA loading buffer, separated by gel electrophoresis in a 3% agarose gel at 100 V for 40 minutes, and visualized under UV light.

### Immunofluorescence *in vivo*

The slides were washed with wash buffer, and heat-induced antigen retrieval was performed using 10 mM sodium citrate with 0.05% Tween 20 (SERVA Electrophoresis) for 30 minutes at 95°C or with 2 N HCl for 30 minutes at 37°C for BrdU staining. After 20 minutes at room temperature, the slides were washed with Tris-buffered saline/Tween 20 (TBST), treated with TBST containing 0.2% Triton X-100 (Carl Roth, Karlsruhe, Germany) and 10% donkey serum (Merck) for 30 minutes, and incubated overnight at 4°C with primary antibodies (Table 1) or Isolectin GS-IB4 From Griffonia simplicifolia (Thermo Fisher Scientific) for vessel staining.

**Table 1:** List of primary antibodies used in this study.

Primary Antibody	Dilution	RRID
Mouse anti-BrdU	1:300	AB_2536432
Mouse anti-Phospho-Histone H3 (Ser10)	1:100	AB_331748
Mouse anti-Satb2	1:500	AB_882455
Rabbit Anti-Cleaved-Caspase-3	1:200	AB_2341188
Rabbit anti-NeuN	1:600	AB_11205760
Rabbit anti-Pax-6	1:300	AB_2749901
Rabbit anti-Tbr1	1:500	AB_2200219
Rabbit anti-Tbr2	1:1000	AB_2721040
Rat anti-Ctip2	1:250	AB_2064130
Rat anti-Tbr2	1:400	AB_11042577

The slides were then incubated with the appropriate secondary antibodies (Thermo Fisher Scientific), and the cell nuclei were stained with Hoechst 33258 (Merck). Finally, the slides were mounted using Fluoromount-G (Biozol, Eching, Germany). It is noteworthy that we were able to achieve sufficient BrdU staining in NesCre-HIF-2α-CKO mice, but unfortunately not in Emx1-HIF-2α-CKO mice, for unknown reasons.

### Neural stem cell isolation, culture, and treatments

Cortices from E14.5 HIF-2α^flox/flox^ and NesCre-HIF-2α CKO mice were dissected, and meninges were removed. Tissues were trypsinized for 10 minutes at 37°C (Trypsin/EDTA 0.05%) and incubated with DNase Ι (0.040 mg/ml) and then minced to obtain a single-cell suspension. The cell suspension was seeded onto PEI-Laminin-coated 6-well cell culture plates (Greiner Bio-One, Kremsmünster, Austria) and 8-well chamber slides (ibidi, Gräfeling, Germany) in expansion medium (67% DMEM, 32% F12 + Glutamax (Thermo Fisher Scientific), 2% B27 (Thermo Fisher Scientific), 1% Anti-Anti (Thermo Fisher Scientific), EGF 20 ng/ml, FGF 20 ng/ml (Thermo Fisher Scientific) and cultured up to 11 days under physioxic (3% O_2_, 5% CO_2_) and hyperoxic conditions (21% O_2_, 5% CO_2_). To ensure constant oxygen tensions, the media used were pre-equilibrated for 24 h. Cells were not passaged during the cultivation period.

### DNA/RNA isolation and qPCR

To obtain DNA/RNA from brain tissue, the cortices of the embryos were isolated at E16.5, homogenized using Precellys^®^ 24 (Bertin Technologies SAS, Montigny-le-Bretonneux, France), and DNA/RNA was isolated using the Qiagen AllPrep DNA/RNA/Protein Mini Kit (QIAGEN, Hilden, Germany) according to the manufacturer’s protocol. To obtain RNA from cultured cells, RNA was isolated using the Macherey-Nagel (Düren, Germany) RNA Isolation NucleoSpin RNA XS kit according to the manufacturer’s instructions. The determined RNA was transcribed into cDNA using the QuantiNova Transcription Kit (QIAGEN). QuantiNova SYBR Green PCR kit (QIAGEN) was used to amplify the subsequent qPCR with 20 ng of cDNA and qPCR primer (Table 2, Eurofins MWG Operon, Ebersberg, Germany) in the following program: Initiation step (2 min, 95°C), 2-step cycling (denaturation: 5s, 95 °C; annealing: 10s, 60°C) with 38 cycles and with a melting curve afterwards. The Delta-delta-ct method was used for quantitative analysis, and Hydroxymethylbilane synthase (*Hmbs*) was used as a housekeeping gene.

**Table 2:** List of primers used in this study.

Target	Forward primer	Reverse primer
*Hmbs*	TGAAATCATTGCTATGTCCACCACG	TCAGGGAGTGAACGACCAGG
*HIF-2α*	CTGAGGAAGGAGAAATCCCGT	TGTGTCCGAAGGAAGCTGATG
*HIF-1α*	TGATGGTGCTAACAGATGACGGC	ACTGGGCCATTTCTGTGTGTAAGC
*Sox2*	TGGACTGCGAACTGGAGAAGG	GCGTTAATTTGGATGGGATTGGTGG
*Vegf*	GCTACTGCCGTCCGATTG	CTCCAGGGCTTCATCGTTAC
*Slc2a1*	GTGACGATCTGAGCTACGGG	CTCCCACAGCCAACATGAGG
*Tcf1*	CCAGTGTGCACCCTTCCTAT	AGCCCCACAGAGAAACTGAA
*Lef1*	AGATCCTGGGCAGAAGATGGC	TGGGACCTGTACCTGAAGTCG
*Hes1*	GTCAACACGACACCGGACAAACC	TCTAGCTTGGAATGCCGGGAGC
*Notch1*	ATTGAGGCCGTGAAGAGTGAGCC	CACAGCCCACAAAGAACAGGAGC
*Olig2*	CAAGAAGCAGATGACTGAGCCCG	GCATGAGGATGTAGTTTCGCGCC
*Map2*	CAGTGGAGGAAGCAGCAAGTGG	GAAGGTCTTGGGAGGGAAGAACG

To verify the knock-out detection at the DNA level, the following primer was used for the *HIF-2α* exon 2 gene (FP: AGAAATCCCGTGATGCCGC, RP: ATGATGGAGGCTTTGTCCAGG), which was normalized to the following HIF-2α reference gene (FP: TCGGTGCGTACTTCCTAGCC, RP: ACCTAGCAGAACCTGACCACG) and also calculated using the delta-delta CT method and internal normalization using a calibrator and in relation to the control cells.

### Protein isolation and western blot

To stabilize the rapidly degrading HIF proteins, cortical NSCs were treated with DMOG (1mM) 6 h before harvest. Cells were scraped on ice as fast as possible and digested using Pierce^TM^ RIPA Buffer (Thermo Fisher Scientific) and Halt^TM^ Protease Inhibitor (Thermo Fisher Scientific). Protein concentration was determined using Pierce BCA Protein Assay Kit (Thermo Fisher Scientific). 50 µg were boiled for 5 minutes at 99°C and placed in an SDS page and electrophoretically separated at a constant 200 V for 42 min. Transfer to a nitrocellulose membrane was performed at a constant 100 V for 1h. For normalization, total protein was detected using REVERT Total Protein Stain Kit (LICORbio, Lincoln, USA) according to the manufacturer’s instructions. For detection, the non-specific binding sites were blocked using WesternBreeze Blocking Solution (Thermo Fisher Scientific) for 3 h and incubated with the Anti-HIF-2-alpha antibody (1:800, AB_11156727) overnight at 4°C, the secondary Goat anti-Rabbit IgG H&L (IRDye® 800CW) antibody (1:2500, AB_2925189) for 3 h and analyzed using Image Lab 6.0 (Bio-Rad, Hercules, USA) software. In parallel, HIF-1α protein detection was attempted by Western blotting under different experimental conditions. For HIF-1α stabilization, either DMOG (1 mM; Calbiochem, San Diego, USA) or MG132 (10 mM; Selleck Chemicals, Houston, USA) was used. Two different primary antibodies were evaluated: an anti-HIF-1α antibody from Abcam (1:200; AB_296474) and an HIF-1α antibody from R&D Systems (1:500; AB_2116983). In addition, both nitrocellulose membranes (LI-COR Biosciences) and PVDF membranes (Bio-Rad) were tested. A commercially available positive control containing HIF-1α isoform 1 (FabGennix, PC-HIFA) was included to assess the detectability of HIF-1α. However, reliable HIF-1α protein detection could not be achieved in the present NSC culture system under any of the tested conditions.

### Immunocytochemistry

Isolated cortical NSCs from E14.5 embryos were seeded on a PEI/laminin-coated 8-well chamber and cultured under both oxygen conditions. After fixation with PFA (4%), cells were permeabilized with Triton X-100 (0.2%), and nonspecific binding sites were blocked with a combination of TBST, donkey serum (2%), and BSA (1%). Subsequent staining was performed with mouse-anti-Nestin (AB_305313; 1:250) and rabbit-anti-ß-Tubulin III (AB_262133; 1:500). The following secondary antibody were used: Donkey anti-Mouse Alexa Fluor™ 488 (AB_141607; 1:250), and Donkey anti-Rabbit Alexa Fluor™ 647 (AB_2536183; 1:250) The cell nuclei were stained with Hoechst 33342 at a dilution of 1:1400. Images were taken with AxioObserver Z1 with Apotome 2 objective using ZEN blue 2.3 software (Carl Zeiss, Oberkochen, Germany).

### Image acquisition and quantification

Immunofluorescence images were acquired using an Axio Observer Z1 microscope (Carl Zeiss) equipped with a 20 × objective and ZEN Blue software, utilizing the “Tiles and Position” module. If not stated otherwise, BrdU ⁺ , NeuN ⁺ , Pax6 ⁺ , Tbr2 ⁺ , and phH3 ⁺ cells were manually counted across Z-stacks from medial cortical sections along the rostro-caudal axis, beginning at the rostral side of the lateral ventricles to the caudal side (that means anterior to the splenium). All quantifications were performed by the same investigator, who was blinded to treatment groups to minimize bias. For cortical layer markers (layer IV neurons: Tbr1, layer V neurons: Ctip2, and upper layer neurons: Satb2), automated cell quantification was performed using ZEN Blue software with the analysis module. Images from at least 4–5 distinct brain sections per animal were included. The boundary between the ventricular zone (VZ) and subventricular zone (SVZ) was determined based on nuclear morphology and the relative density of Pax6⁺ and Tbr2 ⁺ cells: VZ nuclei appeared elongated and densely packed with Pax6 ⁺ cells, whereas SVZ nuclei were elliptical and characterized by higher Tbr2 ⁺ cell density. The counting frame for immunofluorescence analyses was 200 µm in width, except for phH3 ⁺ cells, which were quantified over 500 µm due to their relatively low abundance. For vascularization analysis, binary threshold images were generated from the medial focal plane of each brain section (excluding meningeal areas), and the vascular coverage was calculated as a percentage of the total area. For *in vitro* studies, at least 10 images were randomly acquired from each well. Image analysis was performed using ZEN Blue software with the analysis module. Nestin⁺ neural stem cells were quantified automatically, while βIII-Tubulin⁺ neurons were manually counted.

### Statistical analysis

All statistical analyses were conducted using RGUI 3.4.1 (R Foundation for Statistical Computing, Vienna, Austria) or SPSS version 28.0 (IBM Corp., Armonk, NY, USA). When assumptions of normal distribution and homogeneity of variance were met, either the t-test or two-way ANOVA with pairwise *post-hoc* unpaired two-sided t-test with Bonferroni adjustment were used. If assumptions of normality or equal variances were violated, either the Mann-Whitney U test or robust ANOVA from R (Rfit package) with pairwise *post-hoc* Mann–Whitney U tests with Bonferroni adjustment were applied. In cases of normal distribution but unequal variances, the Welch’s t-test was employed. The number of embryos or pups analyzed is indicated by “n” and represents samples obtained from at least three independent litters. Graphs display mean values ± standard error of the mean (SEM) and were generated using GraphPad Prism 10 (Graphpad Software, Boston, USA).

### Declaration of AI-assisted technologies

During the preparation of this work, the authors used ChatGPT and Grammarly in order to improve the tonality of prewritten text. After using these tools, the author(s) reviewed and edited the content as needed and take(s) full responsibility for the content of the publication.

## Results

### HIF-2α regulates neither Pax6^+^ nor Tbr2^+^ stem/progenitor cell composition of the VZ/SVZ in the developing mouse brain

We initially investigated whether HIF-2α affects the precise pattern of cell division, differentiation, and survival of neuroprogenitors by using Nestin-driven and Emx1-driven HIF-2α CKO models just after the mid-neurogenesis phase at E16.5 (Fig 1a). NesCre- and Emx1-HIF-2α CKO animals, as well as controls, were identified via PCR of generic Cre recombinase (S1a Fig in S1 File). HIF-2α CKO was confirmed by reduced HIF-2α DNA and RNA levels in NesCre-HIF-2α CKO mice under physiological oxygen conditions (S1a Fig in S1 File). Further, HIF-2α protein was reduced after HIF-2α CKO, which was shown in NSCs isolated from NesCre-HIF-2α CKO mice at E14.5 and cultivated *in vitro* under physiological (3% pO_2_) and hyperoxic (21% pO_2_) conditions (S2a Fig in S1 File). While the expression of the domain-related HIF-1α remained unchanged *in vivo* and *in vitro*, HIF-2α downstream genes *Slc2a1* and *Vegf* were solely affected in HIF-2α CKO NSCs *in vitro*, but not by HIF-2α CKO *in vivo* (S1b and S2b Fig in S1 File). Other genes like *Sox2*, which is involved in neural stem cell identity, or *Lef1* and *Tcf1,* associated with WNT signaling pathway, remained unchanged (S1c and S2b Fig in S1 File).

We then investigated the influence of HIF-2α on the stem/progenitor cell composition and analyzed the number of Pax6^+^ and Tbr2^+^ cells, well-established markers for radial glial stem cells and intermediate progenitor cells during cortical development [31–33], in the VZ and the SVZ of E16.5 Emx1-HIF-2α CKO mice (Fig 1b-e) and NesCre-HIF-2α CKO mice (S3 Fig in S1 File). Pregnant mice of each line were maintained under ambient oxygen tension (21% pO_2_) or hypoxic conditions (10% pO_2_) from E14.5 to E16.5 to enhance HIF-2α signaling. To further extend these initial experiments, Emx1-HIF-2α CKO mice were additionally exposed to hyperoxic conditions (75% pO_2_) for 48 h to reduce HIF-2α signaling (Fig 1a) [34]. Fluorescence images displayed a reduced number of Pax6^+^ cells and Tbr2^+^ cells after hypoxia, while we neither detected effects by hyperoxia nor dependence on Emx1-HIF-2α CKO in all oxygen conditions (Fig 1b). Statistical analysis confirmed that oxygen has a significant influence on Pax6^+^, Tbr2^+^, and Pax6^+^/Tbr2^+^ double-positive cells, but only in the SVZ (Fig 1c-e). Consistent with these findings, we could not detect any effect of NesCre-HIF-2α CKO on Pax6^+^ or Tbr2^+^ cells in E16.5 mice, in which only oxygen appears to influence the number of stem and progenitor cells (S3 Fig a,b in S1 File).

Since it was demonstrated in a previous study on NesCre-HIF-2α CKO mice that there is a reduced number of pyramidal neurons within the retrosplenial cortex in the young adult stage at the age of P70 [28], we analyzed whether early Emx1-HIF-2α CKO specifically affects stem/progenitor cell numbers within these retrosplenial areas of the developing cortex (S4 Fig in S1 File). However, we could not detect any effects of HIF-2α CKO or different oxygen tension on Pax6^+^, Tbr2^+,^ or Pax6^+^/Tbr2^+^ cells (S4 Fig a-d in S1 File).

Together, HIF-2α does not regulate stem/progenitor cell composition within the VZ and SVZ in prefrontal and retrosplenial cortical areas of the developing mouse brain at the end of the mid-neurogenesis phase at E16.5, independent of the oxygen tension across hypoxic, physioxic, and hyperoxic conditions.

### HIF-2α CKO shows no effects on proliferation in the developing mouse brain

HIF-2α has been reported to regulate the proliferation of embryonic stem cells but not NSC proliferation, as shown in *in vitro* experiments [22,28,35]. However, we previously observed a differential regulation of proliferation in the developing cortex by oxygen *in vivo,* with decreased proliferation in the SVZ under hypoxia as compared to physioxia (previously named normoxia), independent of HIF-1α [23]. We here investigated whether HIF-2α regulates proliferation by analyzing the number of phH3^+^ mitotic cells in Emx1-HIF-2α CKO mice treated under hypoxic (10% pO_2_), physioxic (21% pO_2_) and hyperoxic (75% pO_2_) conditions (Fig 1b, 1f-1h) and NesCre-HIF-2α CKO mice under hypoxic (10% pO_2_) and physioxic (21% pO_2_) conditions (S3 Fig c in S1 File). phH3 staining showed a decrease in phH3^+^ cells under hypoxic conditions as compared to physioxic conditions, but no effects by hyperoxia (Fig 1b). Quantification revealed main effects of oxygen on the number of phH3^+^ cells in the VZ and the SVZ, however, there was no effect of HIF-2α in Emx1-HIF-2α CKO mice (Fig 1f). We further examined the proliferating stem cell population (double-positive phH3 ⁺ /Pax6⁺) and proliferating progenitor cells (double-positive phH3 ⁺ /Tbr2⁺) and found that only oxygen levels, but not the CKO, influenced their proliferation. Consistently, we did not find any HIF-2α CKO effects in NesCre-HIF-2α CKO mice nor in the retrosplenial area of the developing Emx1-HIF-2α CKO cortex (S3 Fig c, S4 Fig e in S1 File).

Taken together, HIF-2α is not involved in the regulation of cell proliferation within the VZ/SVZ under different conditions.

### HIF-2α does not regulate the number of stem/progenitor cells nor proliferation in the early neurogenesis phase at E13.5

We next investigated the effects of HIF-2α in the early neurogenesis phase of cortical development at E13.5 by using the Emx1-HIF-2α CKO mouse model and again treated them for 48 h with hypoxia (10% pO_2_), physioxia (21% pO_2_), and hyperoxia (75% pO_2_) from E11.5 to E13.5 (Fig 2a). Consistent with our previous study, in which we analyzed Sox2^+^ (which substantially overlaps with Pax6^+^ cells), Tbr2^+^, and phH3^+^ cells in E12.5 embryos [23,36], hyperoxic changes (75% pO_2_) in maternal oxygen tension did not result in any changes of Pax6^+^, Tbr2^+^, or phH3^+^ cells in the E13.5 cortex (Fig 2b-e). In addition, we did not observe any change in the numbers of Pax6^+^, Tbr2^+^, or phH3^+^ cells within the VZ and SVZ in HIF-2α CKO as compared to HIF-2α^flox/flox^ embryos, independent of oxygen condition (Fig 2b-e).

**Fig 2:**
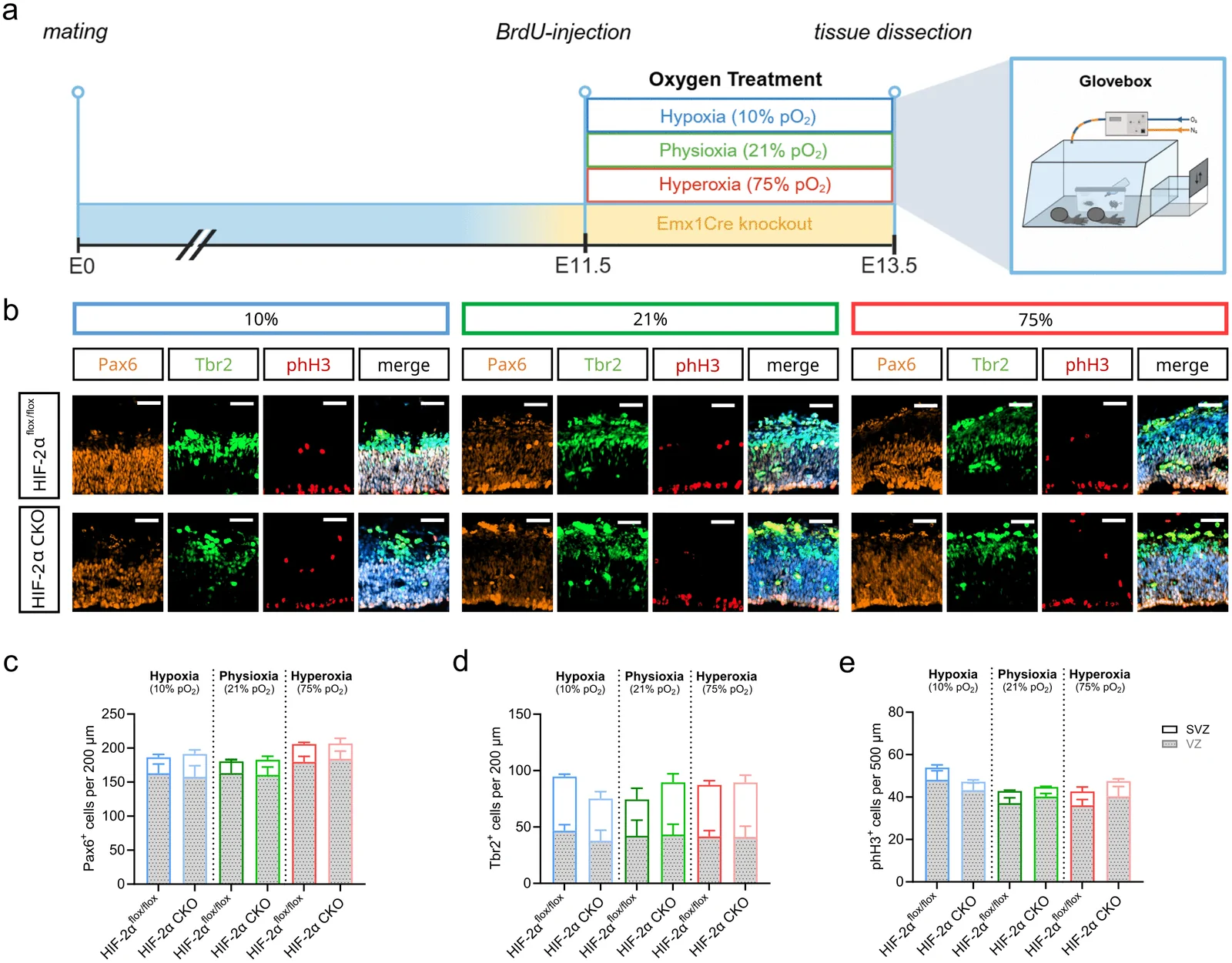
Effects of Emx1-HIF-2α CKO on neural stem/progenitor cell composition and proliferation in the developing mouse cortex at E13.5. (a) Graphical representation of the experimental design. After mice were mated at a defined time-point, pregnant mice received a BrdU injection on E11.5, immediately before maternal oxygen treatment for 48 h with 10% pO_2_, 21% pO_2,_ or 75% pO_2_. Tissue dissection was performed on E13.5. The Emx1-HIF-2α CKO starts at ~E10.5. (b) Representative images of Pax6^+^ neural stem cells, (orange), Tbr2^+^ intermediate progenitor cells (green) and phH3^+^ mitotic cells (red) in the developing cortex of E13.5 HIF-2α^flox/flox^ (top panels) and Emx1-HIF-2α CKO (bottom panels) mice kept under hypoxia (10% pO_2_, left panel), physioxia (21% pO_2_, middle panel) or hyperoxia (75% pO_2_, right panel) for 48 h. Cell nuclei were counterstained with Hoechst (blue). Scale bar = 50 μm. (c-e) Quantification of Pax6 ⁺ cells (c), Tbr2 ⁺ cells (d) per 200 μm, and phH3 ⁺ cells (e) per 500 μm cortical column in E13.5 HIF-2α^flox/flox^ and Emx1-HIF-2α CKO mice kept under the three oxygen conditions. Cells were separately counted in the ventricular zone (VZ; gray with dotted fill) and subventricular zone (SVZ; white). Statistical analysis was performed using two-way ANOVA or robust two-way ANOVA. Data are presented as mean ± s.e.m (n ≥ 4). For full statistics for (c-e), see S7-S9 Tables in S1 File.

### HIF-2α does not affect prenatal cortical mid-neurogenesis

To further assess proliferating cells, pregnant mice were injected with BrdU at E14.5. This allowed us to label cells in S-phase to quantify proliferation and, in parallel, to trace the subsequent fate of these cells toward neuronal differentiation over time. BrdU staining did not yield evaluable results in the Emx1-HIF-2α CKO model under the conditions used. Therefore, mid-neurogenesis was assessed using the NesCre-HIF-2α CKO model, treated with hypoxic oxygen tension (10% pO_2_) compared to physioxic conditions (21% pO_2_), instead. Quantification of the total number of NeuN^+^ neurons and at E14.5 newborn BrdU^+^/NeuN^+^ neurons in the sub-cortical/cortical plate via immunohistochemistry staining showed no significant changes in total NeuN^+^ neurons but a decrease of newborn BrdU^+^/NeuN^+^ neurons in the deeper cortical layer in hypoxic as compared to physioxic E16.5 mouse fetuses (Fig 3), similar as reported previously by Wagenführ and co-workers [23]. However, no effects of HIF-2α CKO on total neurons and newborn neurons in the sub-cortical and cortical plate were detected in both oxygen conditions (Fig 3). In addition, NesCre-HIF-2α CKO showed no effects on vessel density or the number of CC3^+^ apoptotic cells (S5 Fig in S1 File), whereas under hypoxic conditions, a significant increase in CC3 ⁺ cells was observed on the apical side. Despite this hypoxia-induced increase in apoptosis, total NeuN⁺ neuron numbers remained unchanged and were not affected by either oxygen conditions or HIF-2α CKO in the Emx1-HIF-2α CKO model (S6 Fig in S1 File).

**Fig 3:**
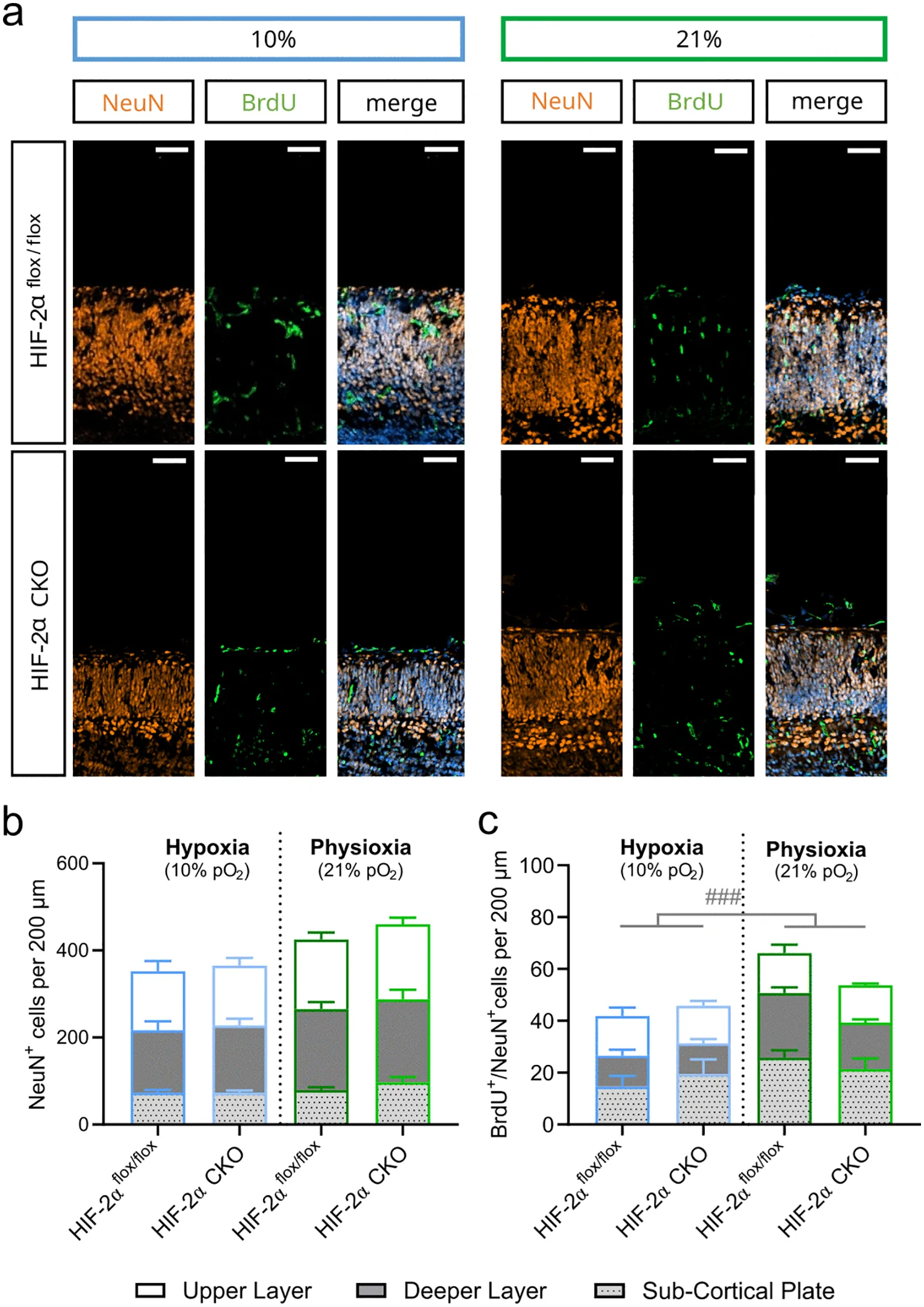
Effects of HIF-2α CKO on prenatal cortical early/mid-neurogenesis in the developing mouse cortex at E16.5. (a) Representative images of newborn cells (BrdU, green) and neurons (NeuN, orange) in cortices of E16.5 HIF-2α^flox/flox^ (top panels) and NesCre-HIF-2α CKO (bottom panels) fetuses exposed to hypoxia (10% pO_2_, left panel) and physioxia (21% pO_2_, right panel) for 48 h. Cell nuclei were counterstained with Hoechst (blue). Scale bar = 50 μm. (b,c) Quantification of NeuN⁺ neurons (b) and newborn BrdU^+^/NeuN⁺ neurons (c) per 200 μm cortical column in E16.5 HIF-2α^flox/flox^ and NesCre-HIF-2α CKO mice kept under hypoxia (10% pO_2_, blue) or physioxia (21% pO_2_, green). Cells were separately counted in the sub-cortical plate (gray with dotted fill), deeper cortical layer (dark gray), and upper cortical layer (white). Data are presented as mean ± s.e.m (n ≥ 4). Statistical analysis was performed using two-way ANOVA or robust two-way ANOVA. # indicates significance in the main effect of oxygen (gray for deeper layer), ### p < 0.001. p-values from *post-hoc* t-test with Bonferroni adjustment are omitted from the diagram for clarity. For full statistics for (b,c), see S10-S11 Tables in S1 File.

### HIF-2α does not alter layering in the developing mouse cortex

Since HIF-2α had no effects on the number of total cortical neurons under both alternate oxygen conditions, we investigated the effects of HIF-2α CKO on neuronal positioning and subtyping within the developing cortex. We thus analyzed specific cortical layering in Emx1-HIF-2α CKO mice as the CKO covers the whole early/mid-neurogenesis phase (effective from ~E10.5) [27,37] (Fig 4). We did neither detect any influence of hypoxia as compared to physioxia nor of HIF-2α CKO on cortical layering, nor on Tbr1^+^ deep layer VI neurons, nor on Ctip2^+^/Tbr1^-^ layer V neurons, nor on Satb2^+^ upper layer neurons (Figs 4a-d). Together, HIF-2α is neither involved in cortical layering under physiological fetal brain oxygen conditions nor under hypoxic conditions known to activate HIF-2α.

**Fig 4:**
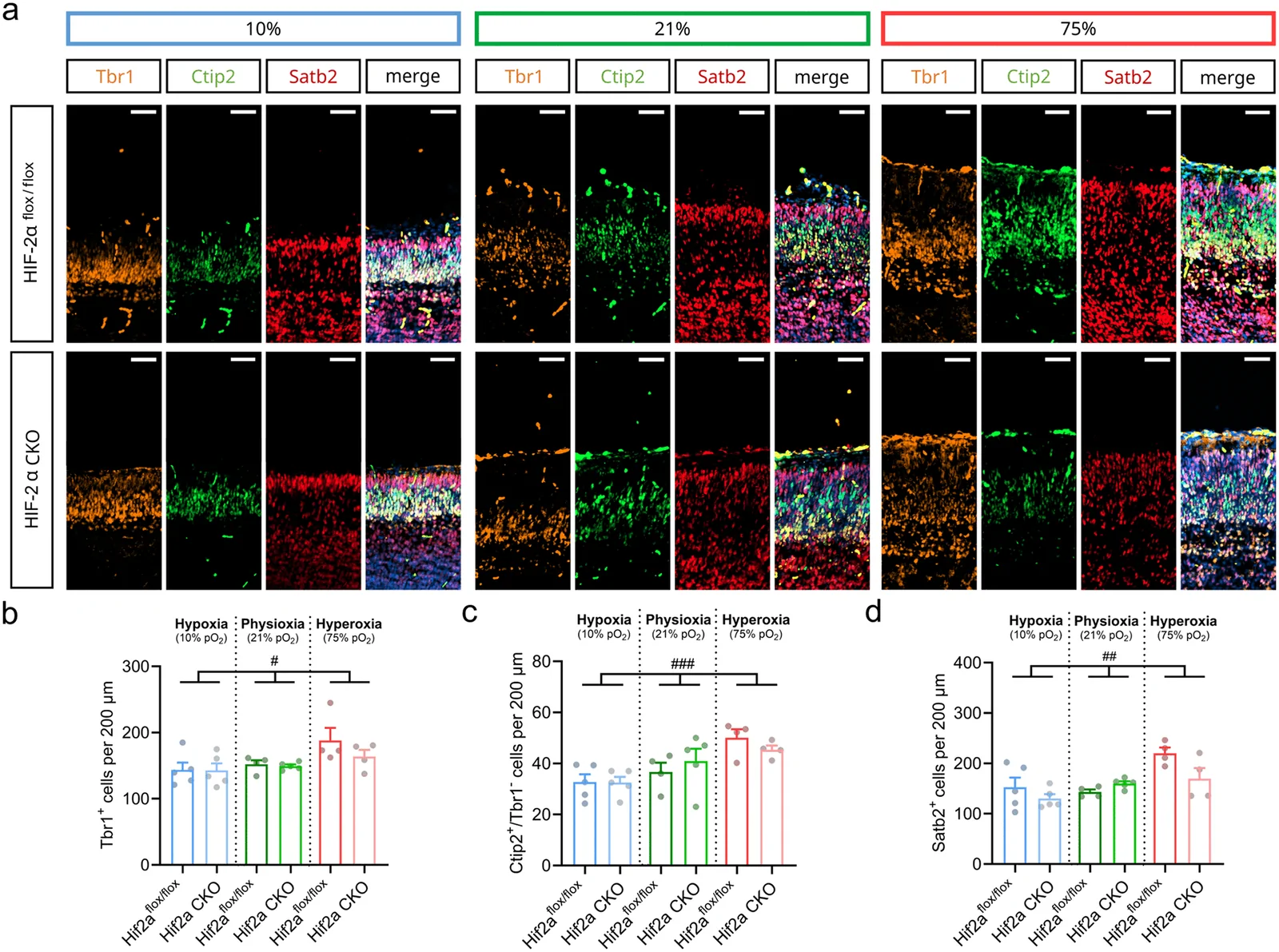
Effects of HIF-2α CKO on cortical layering in the developing mouse cortex at E16.5. (a) Representative images of layer VI neurons (Tbr1, orange), layer V neurons (Ctip2, green) and upper layer neurons (Satb2, red) in E16.5 HIF-2α^flox/flox^ (top panels) and Emx1-HIF-2α CKO (bottom panels) fetuses kept under hypoxia (10% pO_2_, left panel), physioxia (21% pO_2_, middle panel) or hyperoxia (75% pO_2_, right panel) for 48 h. Cell nuclei were counterstained with Hoechst (blue). Scale bar = 50 μm. (b-d) Quantification of Tbr1 ⁺ layer VI neurons (b), Ctip2 ⁺ /Tbr1^-^ layer 5 neurons (c) and Satb2 ⁺ upper layer neurons (d) per 200 μm cortical column in E16.5 HIF-2α^flox/flox^ and Emx1-HIF-2α CKO fetuses kept under hypoxic (10% pO_2_, blue), physioxic (21% pO_2_, green) and hyperoxic (75% pO_2_, red) conditions. Data are presented as mean ± s.e.m (n ≥ 4). Statistical analysis was performed using two-way ANOVA or robust two-way ANOVA. # indicates significance in the main effect of oxygen. # p ≤ 0.05, ## p < 0.01, ### p < 0.001. p-values from *post-hoc* t-test with Bonferroni adjustment are omitted from the diagram for clarity. For full statistics for (b-d) see S12-S14 Tables in S1 File.

### HIF-2α does not influence hyperoxia-induced acceleration of layer V development

In our previous studies, we observed a hyperoxia-induced acceleration of layer V development [23,25], and we therefore sought to determine whether this mechanism is mediated by HIF-2α. Visually, an enlargement of the cortex can be seen with increasing oxygen tension (Fig 4a). Quantified, it is reflected by a slight increase of Tbr1^+^ deep layer VI neurons, Ctip2^+^/Tbr1^-^ layer V neurons, and Satb2^+^ upper layer neurons due to hyperoxia as compared to hypoxia or physioxia (S12-S13 Tables in S1 File). Upon closer examination of layer V cells, we observed an increase in Ctip2^+^ layer V cells in animals treated with hyperoxia from E14.6 to E16.5 (p = 0.045). In contrast, HIF-2α CKO did not show any effect on cortical layering, nor on Tbr1^+^, Ctip2^+^/Tbr1^-^, or Satb2^+^ neurons (Fig 4b-d). Together, hyperoxia is able to increase cortical layering, particularly the number of layer V neurons, but HIF-2α is neither involved in either mechanism.

### HIF-2α does not affect NSC proliferation and stem cell maintenance *in vitro*

To confirm the data on HIF-2α effects on cortical development and on stem/progenitor cells in the developing mouse brain, we analyzed the effects of HIF-2α CKO on proliferation and stem cell maintenance of E14.5 NSCs generated from NesCre-HIF-2α CKO mouse cortex expanded *in vitro* under physioxia (3% pO_2_) and hyperoxia (21% pO_2_) at least for 6 days (Fig 5). The HIF-2α CKO in this E14.5 NSC model was confirmed on the RNA and protein level as well as on the level of downstream gene expression (S2 Fig in S1 File). In contrast to the *in vivo* condition, the physiological oxygen level for NSCs in cell culture experiments is 1–5% depending on the respective location in the developing fetal brain (therefore reduced here down to physioxia of 3% pO_2_), while the oxygen tension of 21% pO_2_, which is commonly used in cell culture experiments, needs to be considered as hyperoxic for fetal NSCs [38,39]. As shown in Fig 5, NSCs under physioxic conditions expanded much faster than NSCs under hyperoxic conditions, but their proliferation was not affected by NesCre-HIF-2α CKO (Fig 5a,b). We additionally checked for differentiation potential and stem cell maintenance of NSCs in dependency of oxygen and HIF-2α CKO (Fig 5c,d): Hyperoxia reduced stem cell maintenance, reflected by a shift from Nestin⁺ stem/progenitor cells towards βIII-tubulin⁺ neuronal cells. In contrast, transcriptional levels of differentiation markers (*Olig2*, *Map2*) and Wnt/Notch-associated self-renewal genes (*Tcf1*, *Lef1*, *Notch1*, *Hes1*) remained unaffected, suggesting that the impaired proliferation and stemness are not mediated through oxygen-dependent regulation of these pathways. Also, the HIF-2α CKO did not provoke any changes in these stem cell properties.

**Fig 5:**
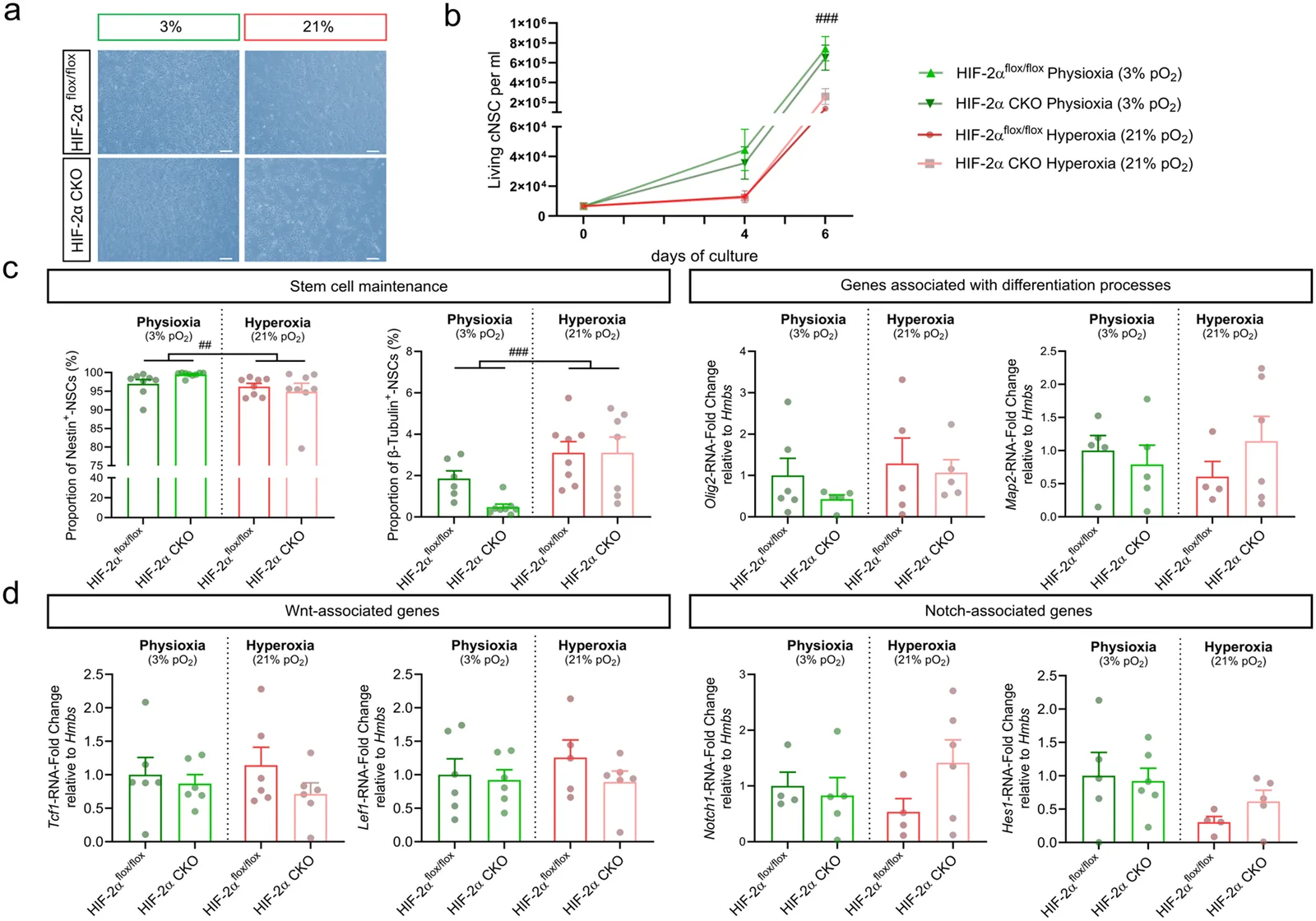
Effects of HIF-2α CKO on proliferation and stem cell maintenance of E14.5 neural stem cells (NSCs) *in vitro.* (a,b) Representative phase-contrast images (a) of cortical NSCs from HIF-2α^flox/flox^ and NesCr-HIF-2α CKO mice cultured under physioxia (3% pO_2_) or hyperoxia (21% pO_2_) for 6 days. Quantification of viable cells (b) was performed at seeding, 4, and 6 days of culture. (c,d) Analysis of spontaneous differentiation after 6 days of culture, cortical NSCs from HIF-2α^flox/flox^ and NesCr-HIF-2α CKO mice under physioxa (3% pO_2_ *in vitro*, green) and hyperoxia (21% pO_2_, red) by quantification of immunhistochemically stained Nestin⁺ and βIII-Tubulin⁺ cells and analysis of gene expression of *Olig2* and *Map2*, assessed by quantitative PCR and normalized to *Hmbs* (c). Expression analysis of Wnt-associated genes (*Tcf1*, *Lef1*) and Notch-associated genes (*Notch1*, *Hes1*), assessed by quantitative PCR and normalized to *Hmbs* (d). Data are presented as mean ± s.e.m (n ≥ 5). Statistical analysis was performed using two-way ANOVA or robust two-way ANOVA. # indicates significance in the main effect of oxygen. ## p < 0.01, ### p < 0.001. p-values from *post-hoc* t-test with Bonferroni adjustment are omitted from the diagram for clarity. For full statistics for (b-d), see S15-S17 Tables in S1 File.

Together, the CKO of HIF-2α in fetal NSCs did not result in any effects on cell proliferation or major stem cell maintenance properties.

## Discussion

The primary finding of our studies is that fetal mouse cortical brain development and neurogenesis depend on appropriate levels of oxygen, but the corresponding oxygen effects are not mediated by hypoxia inducible factor 2α (HIF-2α). We utilized either NesCre- or Emx1-driven CKO mice (HIF-2α CKO) and compared them with controls (HIF-2α^flox/flox^) regarding cortical morphology, stem- and progenitor cell characteristics, and gene expression *in vivo* and *in vitro*. In addition, we used different oxygen conditions to either enhance or inhibit oxygen-sensitive HIF-2α activity [40,41], as the embryonic cortex develops under physiologically low oxygen conditions that are sufficient to permit HIF signaling. Importantly, these paradigms have been shown to effectively alter oxygen availability within the developing cortex, as demonstrated by changes in Hypoxyprobe accumulation in response to maternal hypoxia and hyperoxia [23]. Although our results confirm the influence of oxygen tension on the development of the mouse cortex during the early/mid-neurogenesis phase [23,25], there is no evidence that HIF-2α is involved in the regulation of cortical development under physiological as well as hypoxic and hyperoxic conditions: Stem/progenitor cell composition, proliferation and neurogenesis within the developing cortex as well as the cortical layering is not altered by HIF-2α CKO in mouse cortex during early and mid-neurogenesis phase at E13.5 and E16.5. These data are in full agreement with results in NSCs *in vitro* showing no relevant effects of HIF-2α CKO on proliferation and stem cell maintenance.

Oxygen is crucial for brain development, not only for the required energy supply but also as an important signaling molecule [1–4,23–25,42,43] with direct effects on NSC properties. Cell culture studies on NSCs of various embryonic origins demonstrated that low oxygen levels with consecutive high glycolytic activity prevent NSC differentiation, while increased levels of oxygen switch NSCs towards neuro-glial differentiation [16,44–47]. Direct manipulation of the oxygen tension within the neurogenic niche of the developing brain *in vivo,* without relevant influence on vessel formation by controlling maternal atmospheric oxygen, showed that oxygen signaling is an essential mechanism during mid-neurogenesis for regulating cortical and midbrain neurogenesis by controlling cell proliferation and commitment in regions basal to the SVZ [23–25,39,48,49]. In this context, maternal hyperoxia (75% pO_2_), although not reflecting a physiological condition during fetal development, represents a well-established, non-invasive experimental approach to increase fetal cortical oxygen availability and investigate oxygen-dependent mechanisms of cortical development [23,25]. Together, oxygen tension is nowadays considered a critical factor of the neurogenic niche of the mammalian embryonic brain [50,51].

Previous cell culture studies suggested that oxygen-dependent regulation of NSC behavior is mediated – at least in part – by HIF-1α signaling [14–17]. However, the direct role of HIFs in regulating cortical neurogenesis *in vivo* remains incompletely understood. While early Emx1-driven HIF-1α deletion (~E10.5) altered cortical progenitor composition, with reduced stem cell numbers and increased progenitor cell numbers, and resulted in reduced cortical thickness, these effects were accompanied by severe impairment of brain angiogenesis [2,26]. This makes it difficult to distinguish direct effects on neurogenesis from indirect effects caused by impaired vascular development. In contrast, later NesCre-mediated HIF-1α deletion (~E12.5) caused pronounced vascular defects but only minor changes in stem cell proliferation and did not markedly affect brain growth or cytoarchitecture [23,26]. Collectively, published studies suggest that HIF-1α primarily influences cortical development through angiogenesis, whereas its direct effects on neurogenesis appear to be limited and dependent on early developmental stages [2,23]. Since HIF-2α can activate genes via its hypoxia response element, very similar to HIF-1α [20] but differentially regulates gene expression in a much broader range in various cell types [21,52], HIF-2α is an obvious candidate mediator of oxygen effects in brain development. Indeed, HIF-2α is able to regulate several properties of stem cells, such as proliferation and stemness in various stem cell types, including embryonic stem cells and NSCs [22,35,53–55]. In agreement with the literature [28,39,45,46,56], although high oxygen tension inhibits fetal NSC proliferation and maintenance, loss of HIF-2α in fetal NSCs did not result in any effects on cell proliferation or stem cell maintenance properties under physioxic and hyperoxic conditions. As the most prominent effect on the cellular level, Kleszka and colleagues showed a markedly reduced migration capacity of NSCs isolated at birth by NesCre-HIF-2α CKO [28]. These results are in close agreement with previous results showing that cellular migration is regulated by HIF-2α in various brain-associated tumor cells [57,58] and neural crest cells [18]. Cellular migration is of pivotal importance for cortical layering to form the 6-layered structure of the neocortex [31,59–61].

In agreement with cell culture data, our two knock-out models do not provide any evidence that HIF-2α is involved in the regulation of cortical development under physiological as well as hypoxic or hyperoxic conditions, with no alterations of stem/progenitor cell composition, neurogenesis, and cortical layering by HIF-2α CKO in mouse cortex – including retrosplenial regions – during early and mid-neurogenesis phase at E13.5 and E16.5. We neither detected changes regarding the stem cell pool, progenitor cell pool, nor mitotic activity within the proliferative zone in E13.5 and E16.5 fetuses by HIF-2α CKO. While Pax6 and Tbr2 are well-established and widely accepted markers for assessing the major stem and progenitor cell populations during cortical development [31–33], subtle alterations in molecularly distinct stem or progenitor cell subpopulations cannot be entirely excluded [62–64]. Nevertheless, our findings do not indicate alterations in major stem/progenitor cell populations or in overall cortical neurogenesis. In contrast, Kleszka and colleagues demonstrated that a NesCre-HIF-2α CKO led to a loss of cortical width and pyramidal cells in the retrosplenial cortex as well as altered cognitive functions in young adult (P70) mice [28]. However, the authors assessed the effects of NesCre-HIF-2α CKO only very late after substantial completion of brain development in young adulthood at P70 [28]. The timing and underlying mechanisms of this phenomenon remain unclear and may not necessarily be attributed to impaired fetal cortical brain development.

Given that our data indicate that HIF-2α does not appear to influence early or mid-stage cortical development, and that previous studies reported only mild effects of HIF-1α deficiency on neural stem cells, potentially secondary to its role in angiogenesis [2,23], functional redundancy between HIF-1α and HIF-2α should be considered. Importantly, the hypoxic and hyperoxic conditions used in our study do not selectively modulate HIF-2α, but are expected to affect the activity and stability of both HIF-1α and HIF-2α to varying degrees. Therefore, these oxygen manipulation paradigms cannot be considered selective functional tests of HIF-2α activity, and compensatory HIF-1α signaling may mask potential effects of HIF-2α loss. Although HIF-2α has been reported to regulate a broader set of target genes [20,21,40,52], both transcription factors share overlapping DNA-binding motifs and downstream targets, providing a potential basis for functional compensation in single knock-out models [5,65–67]. Supporting this notion, a neuron-specific HIF-1α/HIF-2α double knock-out model revealed a reduction in Vegf expression in the forebrain of young adult mice following 6 hours of hypoxic exposure, an effect not observed in either single knock-out model [68]. While these findings cannot be directly extrapolated to early or mid-fetal cortical development, they suggest that compensatory mechanisms between HIF-1α and HIF-2α may rescue phenotypes in single knock-out models. A limitation of our study is that we were unable to reliably detect HIF-1α protein under the investigated oxygen conditions despite extensive optimization attempts. Therefore, potential changes in HIF-1α activity and compensatory responses between HIF-1α and HIF-2α cannot be fully excluded. Consequently, potential functional redundancy between these factors should be addressed in future studies.

Taken together, our data indicate that HIF-2α is not critical for fetal cortical brain development during the early/mid-neurogenesis phase under physiological oxygen conditions. Even the application of hypoxia or hyperoxia, which functionally activate/deactivate HIFs, did not lead to any effects on fetal cortical brain development by HIF-2α CKO as compared to HIF-2α^flox/flox^ controls. The systemic data reported by Kleszka and colleagues demonstrating cortical thinning and pyramidal neuron loss in the retrosplenial cortex as well as altered cognitive functions in young adult mice [28] in conjunction with various cell culture data showing HIF-2α-mediated effects on cell migration capacity should actually be interpreted as effects of HIF-2α on later brain development conceivably cortical layering or even a post-developmental phenotype but not as HIF-2α effects during the early or mid-neurogenesis phase of fetal brain development. Future experiments are warranted to further dissect the exact cellular and molecular mechanisms mediating oxygen effects on brain development as a fundamental factor for proper brain development.

## Supporting information

S1 File Supporting Fig. S1-S8 and Supporting Tables S1-S33. (DOCX)

S2 File Raw Western Blot and gel images. (PDF)
