# Supplementary material for: Hypoxia-inducible factor 2α does not regulate cortical brain development
Source: PLoS One. 2026 Oct 14;21(10):e0359507. doi: 10.1371/journal.pone.0359507 (PMC13667805; doi:10.1371/journal.pone.0359507)
Supplement: Supplementary file 1 [file pone.0359507.s002.pdf]

Lane	Sample	Genotyp	Oxygentension [%]
1	Marker		
2	395_Em1	CKO	21
3	395_Em1	CKO	3
4	395_Em2	Flox	21
5	395_Em2	Flox	3
6	395_Em3	Flox	21
7	395_Em3	Flox	3
8	395_Em4	CKO	21
9	395_Em4	CKO	3
10	395_Em5	CKO	21
11	395_Em5	CKO	3
12	397_Em6	Flox	3

Lane	Sample	Genotyp	Oxygentension [%]
1	396_Em5	CKO	21
2	396_Em1	CKO	21
3	396_Em1	CKO	3
4	396_Em2	Flox	3
5	396_Em2	Flox	21
6	Marker		
7	396_Em5	CKO	3
8	x		
9	x		
10	396_Em7	CKO	21
11	396_Em7	CKO	3
12	x		

Lane	Sample	Genotyp	Oxygentension [%]
1	397_Em1	Flox	21
2	397_Em1	Flox	3
3	397_Em2	Flox	21
4	397_Em2	Flox	3
5	397_Em3	Flox	21
6	Marker		
7	397_Em3	Flox	3
8	397_Em4	Flox	21
9	397_Em4	Flox	3
10	397_Em5	Flox	21
11	397_Em5	Flox	3
12	397_Em6	Flox	21

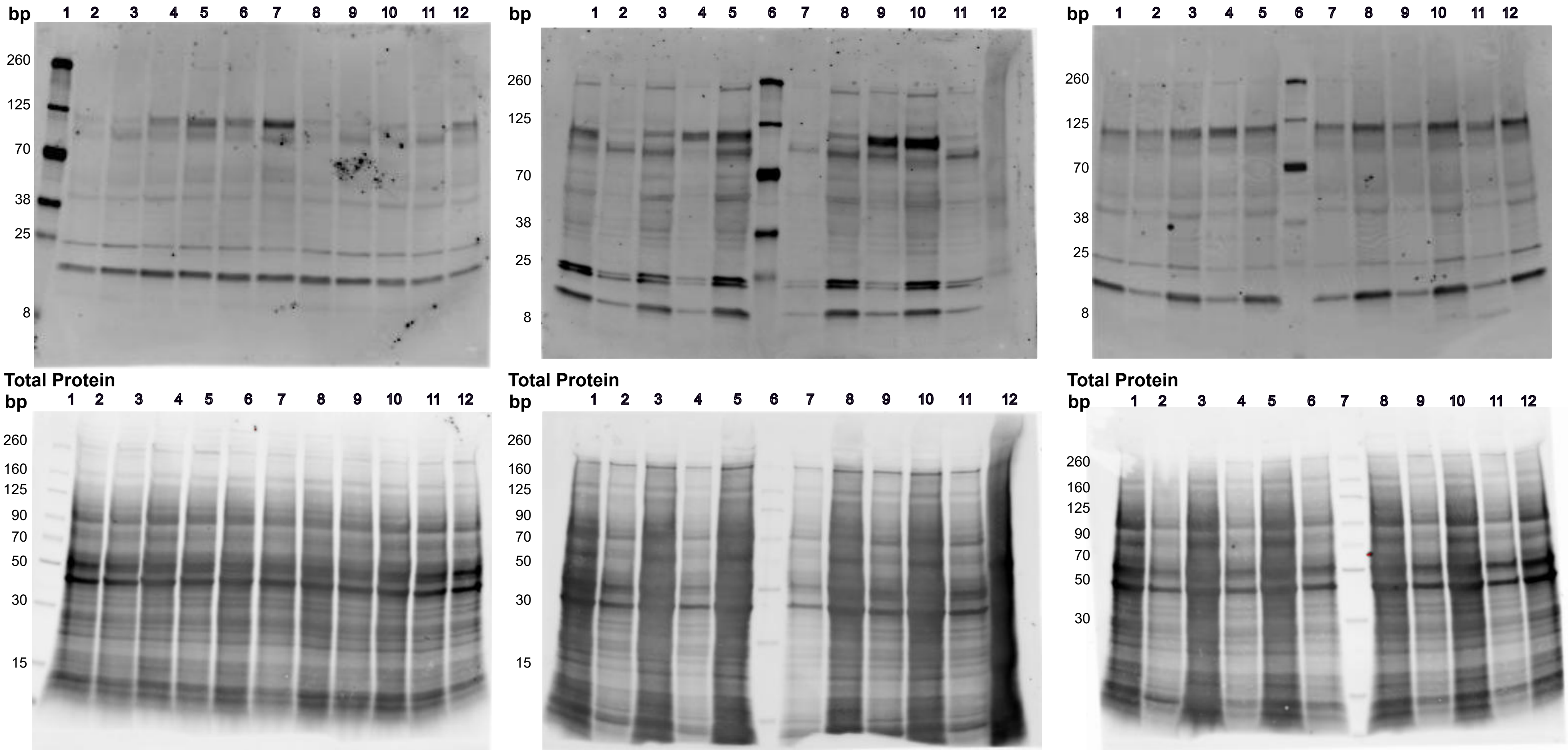

Western blot analysis of HIF-2α protein expression in embryonic brain lysates from HIF-2α conditional knockout (CKO) and floxed control (Flox) mice cultured under 3% or 21% O_2. The tables above each blot indicate the sample assignment, genotype, and oxygen condition. HIF-2α immunoreactivity was detected using a primary anti-HIF-2α antibody (AB_11156727) and imaged in the 800-nm channel. Total protein staining was performed using the Revert™ 700 Total Protein Stain Kit (LI-COR), acquired in the 700-nm channel, and used for normalization of HIF-2α signal intensities. Molecular weight markers are indicated in bp.

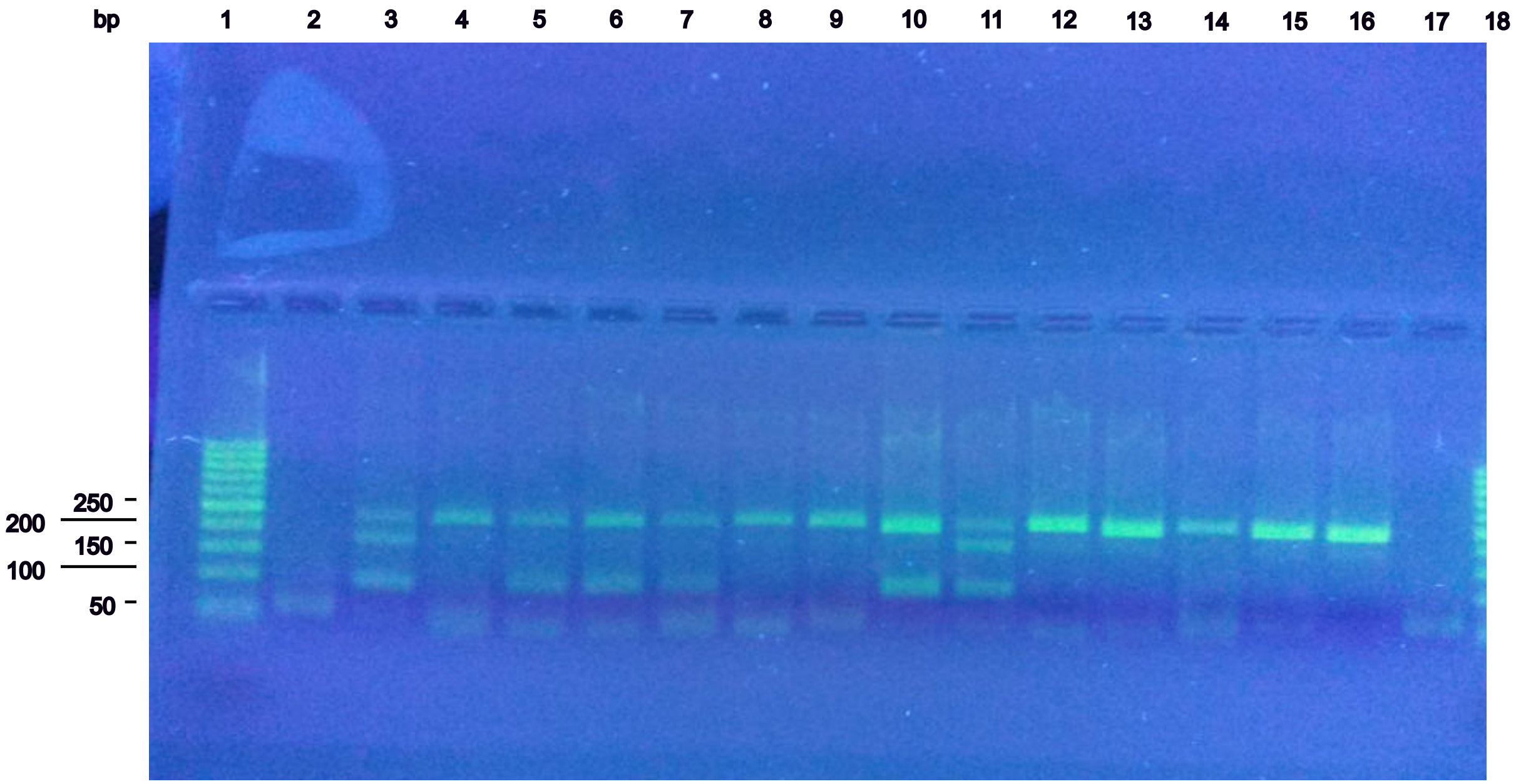

Lane	Sample	Genotype
1	Marker 50 bp	
2	NTC	
3	HIF-2α Positive Control	
4	222_Em7	HIF-2α$^{Flox/Flox}$
5	222_Em6	HIF-2α CKO
6	222_Em5	HIF-2α CKO
7	222_Em4	HIF-2α CKO
8	222_Em3	HIF-2α$^{Flox/Flox}$
9	222_Em2	HIF-2α$^{Flox/Flox}$
10	222_Em1	HIF-2α CKO
11	HIF-2α Positive Control	
12	166_Em5	HIF-2α$^{Flox/Flox}$
13	166_Em4	HIF-2α$^{Flox/Flox}$
14	166_Em3	HIF-2α$^{Flox/Flox}$
15	166_Em2	HIF-2α$^{Flox/Flox}$
16	166_Em1	HIF-2α$^{Flox/Flox}$
17	NTC	
18	Marker 50 bp	

Genotyping of HIF-2α and Cre alleles by PCR. Representative agarose gel showing PCR-based genotyping of HIF-2α and Cre alleles in embryonic mouse samples. A touchdown PCR was performed using following primer: HIF-2α forward primer: GAGAGCAGCTTCTCCTGGAA, reverse primer: TGTAGGCAAGGAAACCAAGG; Cre: forward primer: GCGGTCTGGCAGTAAAAACTATC, reverse primer: GTGAAACAGCATTGCTGTCACTT. PCR products were separated on a 3% agarose gel at 100 V for 40 min. A 50-bp DNA ladder was used as molecular size marker. Lanes 1 and 18 = 50-bp DNA ladder; lanes 2 and 17 = No Template Controls (NTC); lanes 3 and 11, HIF-2α-positive controls. Lane 18 is only partially visible in the original gel image due to the limited image frame. Estimated allele bands: HIF-2α wild-type ~ 180 bp; HIF-2α flox ~ 220 bp; Cre ~ 100 bp
