# Supplementary material for: Hypoxia-inducible factor 2α does not regulate cortical brain development
Source: PLoS One. 2026 Oct 14;21(10):e0359507. doi: 10.1371/journal.pone.0359507 (PMC13667805; doi:10.1371/journal.pone.0359507)
Supplement: Supplementary file 2 [file pone.0359507.s001.docx]

Supporting Information

**Hypoxia-inducible factor 2α does not regulate**

**cortical brain development**

**Jennifer Lanto, Laura Rohmann, Tim Theege, Valeriia Abramenko, Lisa Große, Andreas Madlener, Julia Anna Elisabeth Neitzel, Alexander Storch_,_ Franz Markert***

***Correspondence:** Franz Markert: franz.markert@med.uni-rostock.de

## Supporting Figures:

## Supporting Fig S1: Gene expression profiles of NesCre HIF-2α knockout model *in vivo*.

**Supporting Fig S2:** Gene expression profiles of NesCre HIF-2α knockout model in E14.5 cortical neural stem cells (NSCs) *in vitro*.

**Supporting Fig S3:** Effects of NesCre-HIF-2α CKO on stem/progenitor cell composition in the developing cortex of E16.5 mice.

**Supporting Fig S4:** Effects of Emx1-HIF-2α CKO on stem/progenitor cell composition and proliferation in the retrosplenial cortex of E16.5 mice.

**Supporting Fig S5:** Effects of HIF-2α CKO on prenatal mature neurons in the developing Emx1-Cre mouse cortex at E16.5.

**Supporting Fig S6:** Effects of HIF-2α CKO on vascularization and apoptosis in the developing mouse cortex at E16.5.

## Supporting Tables:

**Supporting Table S1**: Statistics determined for Pax6^+^ cells per 200 µm column of E16.5 Emx1-Cre driven HIF-2α CKO mouse cortices exposed to different oxygen conditions (**Fig 1c**).

**Supporting Table S2:** Statistics determined for Tbr2^+^ cells per 200 µm column of E16.5 Emx1-Cre driven HIF-2α CKO mouse cortices exposed to different oxygen conditions (**Fig 1d**).

## Supporting Table S3: Statistics determined for Pax6^+^/Tbr2^+^ cells per 200 µm column of E16.5 Emx1-Cre driven HIF-2α CKO mouse cortices exposed to different oxygen conditions (Fig 1e).

## Supporting Table S4: Statistics determined for phH3^+^ cells per 500 µm column of E16.5 Emx1-Cre driven HIF-2α CKO mouse cortices exposed to different oxygen conditions (Fig 1f).

## Supporting Table S5: Statistics determined for phH3^+^/Pax6^+^ cells per 200 µm column of E16.5 Emx1-Cre driven HIF-2α CKO mouse cortices exposed to different oxygen conditions (Fig 1g).

## Supporting Table S6: Statistics determined for phH3^+^/Tbr2^+^ cells per 200 µm column of E16.5 Emx1-Cre driven HIF-2α CKO mouse cortices exposed to different oxygen conditions (Fig 1h).

## Supporting Table S7: Statistics determined for Pax6^+^ cells per 200 µm column of E13.5 Emx1-Cre driven HIF-2α CKO mouse cortices exposed to different oxygen conditions (Fig 2c).

## Supporting Table S8: Statistics determined for Tbr2^+^ cells per 200 µm column of E13.5 Emx1-Cre driven HIF-2α CKO mouse cortices exposed to different oxygen conditions (Fig 2d).

## Supporting Table S9: Statistics determined for phH3^+^ cells per 500 µm column of E13.5 Emx1-Cre driven HIF-2α CKO mouse cortices exposed to different oxygen conditions (Fig 3e).

## Supporting Table S10: Statistics determined for NeuN^+^ cells per 200 µm column of E16.5 Nestin-Cre driven HIF-2α CKO mouse cortices exposed to different oxygen conditions (Fig 3b).

## Supporting Table S11: Statistics determined for BrdU^+^/NeuN^+^ cells per 200 µm column of E16.5 Nestin-Cre driven HIF-2α CKO mouse cortices exposed to different oxygen conditions (Fig 3c).

## Supporting Table S13: Statistics determined for Tbr1^+^ cells per 200 µm column of E16.5 Emx1-Cre driven HIF-2α CKO mouse cortices exposed to different oxygen conditions (Fig 4b).

## Supporting Table S14: Statistics determined for Ctip2^+^/Tbr1^-^ cells per 200 µm column of E16.5 Emx1-Cre driven HIF-2α CKO mouse cortices exposed to different oxygen conditions (Fig 4c).

## Supporting Table S15: Statistics determined for Satb2^+^ cells per 200 µm column of E16.5 Emx1-Cre driven HIF-2α CKO mouse cortices exposed to different oxygen conditions (Fig 4d).

## Supporting Table S16: Statistics determined for NSC counts in vitro derived from E14.5 NesCr-HIF-2α CKO and respective control mouse cortices cultures under physioxia (3% pO_2_) and hyperoxia (21% pO_2_) for up to 6 days (Fig 5b).

## Supporting Table S17: Statistics determined for Nestin+ and β-Tubulin^+^ cells (% of total cells) and gene expression of Olig2 and Map2 in NSC cultures in vitro derived from E14.5 NesCr-HIF-2α CKO and respective control mouse cortices cultures under physioxia (3% pO_2_) and hyperoxia (21% pO_2_; Fig 6c).

## Supporting Table S18: Statistics determined for gene expression of *Tcf1*, *Lef1*, *Notch1,* and *Hes1* in NSC cultures *in vitro* derived from E14.5 NesCr-HIF-2α CKO and respective control mouse cortices cultures under physioxia (3% pO_2_) and hyperoxia (21% pO_2_; Fig 6d).

## Supporting Table S19: Statistics determined for *HIF-2α* expression on the DNA- and mRNA level *in vivo* of E16.5 Nestin-Cre driven HIF-2α CKO mouse cortices exposed to physioxia (21% pO_2_; Supporting Fig S1a).

## Supporting Table S20: Statistics determined for *Slc2a1*, *Vegf,* and *Sox2* expression on the mRNA level *in vivo* of E16.5 Nestin-Cre driven HIF-2α CKO mouse cortices exposed to physioxia (21% pO_2_; Supporting Fig S1b).

## Supporting Table S21: Statistics determined for *HIF-1α*, *Lef1,* and *Tcf1* expression on the mRNA level *in vivo* of E16.5 Nestin-Cre driven HIF-2α CKO mouse cortices exposed to physioxia (21% pO_2_; Supporting Fig S1c).

## Supporting Table S22: Statistics determined for expression of HIF-2α-Protein, -DNA and -mRNA level in NSC cultures *in vitro* derived from E14.5 NesCr-HIF-2α CKO and respective control mouse cortices cultures under physioxia (3% pO_2_, green) and hyperoxia (21% pO_2_, red; Supporting Fig S2a).

## Supporting Table S23: Statistics determined for gene expression of *Slc2a1*, *Vegf, Sox2,* and *HIF-1α* in NSC cultures *in vitro* derived from E14.5 NesCr-HIF-2α CKO and respective control mouse cortices cultures under physioxia (3% pO_2_, green) and hyperoxia (21% pO_2_, red; Supporting Fig S2b,c).

## Supporting Table S24: Statistics determined for Pax6^+^ cells per 200 µm column of E16.5 NesCr-HIF-2α mouse cortices exposed to different oxygen conditions (Supporting Fig S3a).

## Supporting Table S25: Statistics determined for Tbr2^+^ cells per 200 µm column of E16.5 NesCr-HIF-2α mouse cortices exposed to different oxygen conditions (Supporting Fig S3b).

## Supporting Table S26: Statistics determined for phH3^+^ cells per 500 µm column of E16.5 NesCr-HIF-2α mouse cortices exposed to different oxygen conditions (Supporting Fig S3c).

## Supporting Table S27: Statistics determined for Pax6^+^ cells per 200 µm column of E16.5 Emx1-Cre driven HIF-2α CKO mouse retrosplenial cortices exposed to different oxygen conditions (Supporting Fig S4b).

## Supporting Table S28: Statistics determined for Tbr2^+^ cells per 200 µm column of E16.5 Emx1-Cre driven HIF-2α CKO mouse retrosplenial cortices exposed to different oxygen conditions (Supporting Fig S4c).

## Supporting Table S29: Statistics determined for Pax6^+^/Tbr2^+^ cells per 200 µm column of E16.5 Emx1-Cre driven HIF-2α CKO mouse retrosplenial cortices exposed to different oxygen conditions (Supporting Fig S4d).

## Supporting Table S30: Statistics determined for phH3^+^ cells per 500 µm column of E16.5 Emx1-Cre driven HIF-2α CKO mouse retrosplenial cortices exposed to different oxygen conditions (Supporting Fig S4e).

## Supporting Table S31: Statistics determined for NeuN^+^ cells per 200 µm column of E16.5 Emx1-Cre driven HIF-2α CKO mouse cortices exposed to different oxygen conditions (Supporting Fig S5b).

## Supporting Table S32: Statistics determined for vessel density within the cortical area of E16.5 Nestin-Cre driven HIF-2α CKO mouse cortices exposed to different oxygen conditions (Supporting Fig S6c).

## Supporting Table S33: Statistics determined for apoptotic CC3^+^ cells within the cortical area of E16.5 Nestin-Cre driven HIF-2α CKO mouse cortices exposed to different oxygen conditions (Supporting Fig S6d).

**S1 Fig. Gene expression profiles of NesCre HIF-2α knockout model *in vivo*. (a)** HIF‑2α CKO verification: (left) Representative genotyping gel, which was used for screening HIF-2α CKO in different breeding NesCre and Emx1 mice. PCR products at ~180 bp indicate wildtype alleles; ~220 bp products indicate floxed alleles; ~100 bp products indicate an inserted Cre recombinase. Resulting genotypes are shown above each lane. NTC = no template control, wt = wildtype allele. (middle) Quantitative analysis of HIF-2α critical exon 2 within the DNA of E16.5 HIF‑2α^flox/flox^ and NesCre-HIF-2α CKO. Data were normalized by reference sequence within the hind parts of the HIF-2α gene. (left) Quantitative analysis of HIF-2α RNA of E16.5 HIF 2α^flox/flox^ and NesCre-HIF-2α CKO relative to *Hmbs*. **(b)** Gene expression analysis of the downstream genes (*Slc2a1*, *Vegf*) and *HIF-1α* in E16.5 HIF‑2α^flox/flox^ and NesCre-HIF-2α CKO *in vivo*, which are not affected by the HIF-2α CKO. **(c)** The gene expression of stemness marker (*Sox2*) and the Wnt-signalling pathway via *Lef1* and *Tcf1* is also not affected by the NesCre-HIF-2α CKO in E16.5 mice. Data in the graphs are presented as mean ± s.e.m (n≥4). Statistical analysis was performed using unpaired *t*-tests or Mann-Whitney U-test, *** p < 0.001. For full statistics for **(a-c)**, see **S18-S20 Tables**.

**S2 Fig. Gene expression profiles of NesCre HIF-2α knockout model in E14.5 cortical neural stem cells (NSCs) *in vitro*. (a)** Quantitative evaluation of western blots (left) of DMOG stabilized HIF-2α protein normalized to total protein confirmed the NesCre HIF-2α CKO in E14.5 NSCs cultivated in physiological oxygen conditions (3% pO_2_, green) and hyperoxia (21% pO_2_, red). (middle/right) Quantitative analysis of *HIF-2α*- and *HIF-1α-*RNA of HIF‑2α^flox/flox^ and NesCre-HIF-2α CKO in E14.5 NSCs cultivated for 6 days in physiological oxygen conditions (3% pO_2_, green) and hyperoxia (21% pO_2_, red). Data were normalized by *Hmbs*. **(b)** Gene expression analysis of the downstream genes (*Slc2a1*,*Vegf*) and *Sox2* of HIF‑2α^flox/flox^ and NesCre-HIF-2α CKO in E14.5 NSCs cultivated for 6 days in physiological oxygen conditions (3% pO_2_, green) and hyperoxia (21% pO_2_, red), normalized by *Hmbs*. **(a,b)** Data are presented as mean ± s.e.m. (n≥4). Statistical analysis was performed using two-way ANOVA or robust two-way ANOVA. # indicates significance in the main effect of oxygen. ### *p* < 0.001. * indicates significance in the main effect of the genotype, * p < 0.05, ** p < 0.01, *** p < 0.001. p-values from *post-hoc* t-test with Bonferroni adjustment are omitted from the diagram for clarity. For full statistics, see **S21-S22 Tables**.

**S3 Fig. Effects of NesCre-HIF-2α CKO on stem/progenitor cell composition in the developing cortex of E16.5 mice. (a-c)** Quantification of Pax6⁺ cells **(a)**, Tbr2⁺ cells **(b)** per 200 μm and phH3⁺ cells **(c)** per 500 μm cortical column in HIF-2α^flox/flox^ and NesCr-HIF-2α CKO mice kept under hypoxia (10% pO₂, blue) and physioxia (21% pO₂, green) for 48 h (refer to **Fig 1a** for experimental design). Cells were separately counted in the ventricular zone (VZ; gray with dotted fill) and subventricular zone (SVZ; white). Data are presented as mean ± s.e.m (n ≥ 3). Statistical analysis was performed using two-way ANOVA or robust two-way ANOVA. # indicates significance in the main effect of oxygen (gray for VZ, black for SVZ), # p ≤ 0.05. For full statistics, see **S23-S25 Tables**.

**S4 Fig. Effects of Emx1-HIF-2α CKO on stem/progenitor cell composition and proliferation in the retrosplenial cortex of E16.5 mice.** **(a)** Representative images of neural stem cells (Pax6, orange), intermediate progenitor cells (Tbr2, green) and mitotic cells (phH3, red) in caudal cortices of HIF-2α^flox/flox^ (top panels) and Emx1-HIF-2α CKO mice (bottom panels) kept under hypoxia (10% pO₂, left panel), physioxia (21% pO₂, middle panel) or hyperoxia (75% pO₂, right panel) for 48 h (refer to **Fig 1a** for experimental design). Cell nuclei were counterstained with Hoechst (blue). Scale bar = 50 μm. **(b-e)** Quantification of Pax6⁺ cells **(b)**, Tbr2⁺ cells **(c)** and Pax6⁺/Tbr2⁺ cells **(d)** per 200 μm and phH3⁺ cells **(e)** per 500 μm cortical column in HIF-2α^flox/flox^ and Emx1-HIF-2α CKO mice kept under hypoxia (10% pO₂, blue), physioxia (21% pO₂, green) or hyperoxia (75% pO₂, red) for 48 h. Cells were separately counted in the ventricular zone (VZ; gray with dotted fill) and subventricular zone (SVZ; white). Data are presented as mean ± s.e.m (n≥4). Statistical analysis was performed using two-way ANOVA or robust two-way ANOVA. No significant main effects of oxygen or genotype were observed. For full statistics, see **S26-S29 Tables**.

**S5 Fig. Effects of HIF-2α CKO on vascularization and apoptosis in the developing mouse cortex at E16.5.** Representative images of vessels (IB4, green, left side) **(a)** and apoptotic cells (CC3, red, right side) **(b)** in cortices of E16.5 HIF-2α^flox/flox^ (top panels) and NesCre-HIF-2α CKO (bottom panels) fetuses exposed to hypoxia (10% pO₂, left panel) and physioxia (21% pO₂, right panel) for 48 h in cortical plate and apical region. Cell nuclei were counterstained with Hoechst (blue). Scale bar for IB4 images = 50 μm and for CC3 = 10 µm. **(c)** Quantitative analysis of vessel density as proportion of the whole cortical area in E16.5 HIF-2α^flox/flox^ and NesCre-HIF-2α CKO fetuses kept under hypoxia (10% pO₂, blue) or physioxia (21% pO₂, green) for 48 h. **(d)** Quantification of apoptotic CC3^+^ cells in the cortex per field of E16.5 HIF‑2α^flox/flox^ and NesCre-HIF-2α CKO fetuses kept under hypoxia (10% pO₂, blue) or physioxia (21% pO₂, green) for 48 h. The cells were counted separately in the cortical plate (gray with tailed dots) and apical side (white). Data are presented as mean ±  s.e.m. (n≥3). Statistical analysis was performed using two-way ANOVA. . # indicates significance in the main effect of oxygen (black for apical side), # p ≤ 0.05. For full statistics for **(c,d)**, see **S30-S31 Tables**.

**S6 Fig. Effects of HIF-2α CKO on prenatal mature neurons in the developing Emx1-Cre mouse cortex at E16.5.** **(a)** Representative images of neurons (NeuN, orange) in cortices of HIF‑2α^flox/flox^ (top panels) and Emx1-HIF-2α CKO mice (bottom panels) kept under hypoxia (10% pO₂, left panel), physioxia (21% pO₂, middle panel) or hyperoxia (75% pO₂, right panel) for 48 h (refer to **Fig 1a** for experimental design). Cell nuclei were counterstained with Hoechst (blue). Scale bar = 50 μm. **(b)** Quantification of NeuN⁺ neurons per 200 μm cortical column in E16.5 HIF-2α^flox/flox^ and Emx1-HIF-2α CKO mice kept under hypoxia (10% pO₂, blue) or physioxia (21% pO₂, green). Cells were separately counted in the sub-cortical plate (gray with dotted fill), the deeper cortical layer (dark gray), and the upper cortical layer (white). Data are presented as mean ± s.e.m (n ≥ 4). Statistical analysis was performed using two-way ANOVA or robust two-way ANOVA. No significant main effects of oxygen or genotype were observed. For full statistics for **(b)**, see **S32 Table**.

**S1 Table. Statistics determined for Pax6^+^ cells per 200 µm column of E16.5 Emx1-Cre driven HIF-2α CKO mouse cortices exposed to different oxygen conditions (Fig 1c).** Cells were counted and analyzed separately for each zone (SVZ = Subventricular Zone, VZ = Ventricular Zone). **(a)** Main effects of oxygen and genotype, as well as interaction effects, on Pax6^+^ cells in the SVZ and the VZ were determined by two-way ANOVA. **(b)** Effects of genotype within each oxygen condition and of oxygen within each genotype were assessed using *post-hoc t*-tests. Displayed are Bonferroni-adjusted *p*-values. Bold values indicate significant differences.

**(a)**

|  | **Main effects of Oxygen** | | **Main effects of Genotype** | | **Interaction effects between Oxygen and Genotype** | |
| --- | --- | --- | --- | --- | --- | --- |
| **SVZ** | F=3.828 | **p=0.035** | F=1.114 | p=0.301 | F=0.429 | p=0.656 |
| **VZ** | F=0.094 | p=0.911 | F=0.067 | p=0.797 | F=0.698 | p=0.507 |

**(b)**

|  | | **Hypoxia** | **Physioxia** | **Hyperoxia** |
| --- | --- | --- | --- | --- |
| HIF-2α^flox/flox^ vs. HIF-2α CKO | **SVZ** | 0.218 | 0.800 | 0.840 |
|  | **VZ** | 0.727 | 0.465 | 0.404 |

|  | **SVZ** | | **VZ** | |
| --- | --- | --- | --- | --- |
|  | **HIF-2α^flox/flox^** | **HIF-2α CKO** | **HIF-2α^flox/flox^** | **HIF-2α CKO** |
| Hypoxia vs. Physioxia | 1.000 | 0.576 | 1.000 | 1.000 |
| Hypoxia vs. Hyperoxia | 0.573 | 0.060 | 1.000 | 1.000 |
| Physioxia vs. Hyperoxia | 0.918 | 0.954 | 1.000 | 1.000 |

**S2 Table. Statistics determined for Tbr2^+^ cells per 200 µm column of E16.5 Emx1-Cre driven HIF-2α CKO mouse cortices exposed to different oxygen conditions (Fig 1d).** Cells were counted and analyzed separately for each zone (SVZ = Subventricular Zone, VZ = Ventricular Zone). **(a)** Main effects of oxygen and genotype, as well as interaction effects, on Tbr2^+^ cells in the SVZ and the VZ were determined by two-way ANOVA. **(b)** Effects of genotype within each oxygen condition and of oxygen within each genotype were assessed using post-hoc *t*-tests. Displayed are Bonferroni-adjusted *p*-values. Bold values indicate significant differences.

**(a)**

|  | **Main effects of Oxygen** | | **Main effects of Genotype** | | **Interaction effects between Oxygen and Genotype** | |
| --- | --- | --- | --- | --- | --- | --- |
| **SVZ** | F=5.457 | **p=0.011** | F=0.443 | p=0.512 | F=0.179 | p=0.837 |
| **VZ** | F=0.849 | p=0.440 | F=0.080 | p=0.780 | F=0.540 | p=0.589 |

**(b)**

|  | | **Hypoxia** | **Physioxia** | **Hyperoxia** |
| --- | --- | --- | --- | --- |
| HIF-2α^flox/flox^ vs. HIF-2α CKO | **SVZ** | 0.423 | 0.959 | 0.811 |
|  | **VZ** | 0.823 | 0.587 | 0.340 |

|  | **SVZ** | | **VZ** | |
| --- | --- | --- | --- | --- |
|  | **HIF-2α^flox/flox^** | **HIF-2α CKO** | **HIF-2α^flox/flox^** | **HIF-2α CKO** |
| Hypoxia vs. Physioxia | 1.000 | 0.075 | 1.000 | 0.654 |
| Hypoxia vs. Hyperoxia | 0.255 | 0.072 | 1.000 | 1.000 |
| Physioxia vs. Hyperoxia | 1.000 | 1.000 | 1.000 | 0.903 |

**S3 Table. Statistics determined for Pax6^+^/Tbr2^+^ cells per 200 µm column of E16.5 Emx1-Cre driven HIF-2α CKO mouse cortices exposed to different oxygen conditions (Fig 1e).** Cells were counted and analyzed separately for each zone (SVZ = Subventricular Zone, VZ = Ventricular Zone). **(a)** Main effects of oxygen and genotype, as well as interaction effects, on Pax6^+^/Tbr2^+^ cells in the SVZ and the VZ were determined by two-way ANOVA. **(b)** Effects of genotype within each oxygen condition and of oxygen within each genotype were assessed using post-hoc *t*-tests. Displayed are Bonferroni-adjusted *p*-values. Bold values indicate significant differences.

**(a)**

|  | **Main effects of Oxygen** | | **Main effects of Genotype** | | **Interaction effects between Oxygen and Genotype** | |
| --- | --- | --- | --- | --- | --- | --- |
| **SVZ** | F=3.894 | **p=0.034** | F=1.499 | p=0.232 | F=0.320 | p=0.729 |
| **VZ** | F=3.138 | p=0.061 | F=0.142 | p=0.710 | F=0.855 | p=0.437 |

**(b)**

|  | | **Hypoxia** | **Physioxia** | **Hyperoxia** |
| --- | --- | --- | --- | --- |
| HIF-2α^flox/flox^ vs. HIF-2α CKO | **SVZ** | 0.219 | 0.828 | 0.553 |
|  | **VZ** | 0.729 | 0.484 | 0.241 |

|  | **SVZ** | | **VZ** | |
| --- | --- | --- | --- | --- |
|  | **HIF-2α^flox/flox^** | **HIF-2α CKO** | **HIF-2α^flox/flox^** | **HIF-2α CKO** |
| Hypoxia vs. Physioxia | 1.000 | 0.432 | 0.852 | 0.150 |
| Hypoxia vs. Hyperoxia | 0.396 | 0.081 | 0.198 | 0.720 |
| Physioxia vs. Hyperoxia | 0.837 | 1.000 | 1.000 | 0.903 |

**S4 Table. Statistics determined for phH3^+^ cells per 500 µm column of E16.5 Emx1-Cre driven HIF-2α CKO mouse cortices exposed to different oxygen conditions (Fig 1f).** Cells were counted and analyzed separately for each zone (SVZ = Subventricular Zone, VZ = Ventricular Zone). **(a)** Main effects of oxygen and genotype, as well as interaction effects, on phH3^+^ cells in the SVZ and the VZ were determined by two-way ANOVA. **(b)** Effects of genotype within each oxygen condition and of oxygen within each genotype were assessed using post-hoc *t*-tests. Displayed are Bonferroni-adjusted *p*-values. Bold values indicate significant differences.

**(a)**

|  | **Main effects of Oxygen** | | **Main effects of Genotype** | | **Interaction effects between Oxygen and Genotype** | |
| --- | --- | --- | --- | --- | --- | --- |
| **SVZ** | F=23.353 | **p<0.001** | F=0.268 | p=0.610 | F=0.245 | p=0.785 |
| **VZ** | F=4.440 | **p=0.024** | F=0.486 | p=0.493 | F=2.543 | p=0.101 |

**(b)**

|  | | **Hypoxia** | **Physioxia** | **Hyperoxia** |
| --- | --- | --- | --- | --- |
| HIF-2α^flox/flox^ vs. HIF-2α CKO | **SVZ** | 0.413 | 0.974 | 0.966 |
|  | **VZ** | 0.316 | 0.792 | **0.037** |

|  | **SVZ** | | **VZ** | |
| --- | --- | --- | --- | --- |
|  | **HIF-2α^flox/flox^** | **HIF-2α CKO** | **HIF-2α^flox/flox^** | **HIF-2α CKO** |
| Hypoxia vs. Physioxia | **0.015** | 0.156 | **0.036** | 0.609 |
| Hypoxia vs. Hyperoxia | **<0.001** | **<0.001** | 0.123 | 0.987 |
| Physioxia vs. Hyperoxia | 0.093 | 0.129 | 1.000 | 0.078 |

**S5 Table. Statistics determined for phH3^+^/Pax6^+^ cells per 200 µm column of E16.5 Emx1-Cre driven HIF-2α CKO mouse cortices exposed to different oxygen conditions (Fig 1g).** Cells were counted and analyzed separately for each zone (SVZ = Subventricular Zone, VZ = Ventricular Zone). **(a)** Main effects of oxygen and genotype, as well as interaction effects, on phH3^+^/Pax6^+^ cells in the SVZ and the VZ were determined by two-way ANOVA. **(b)** Effects of genotype within each oxygen condition and of oxygen within each genotype were assessed using post-hoc *t*-tests. Displayed are Bonferroni-adjusted *p*-values. Bold values indicate significant differences.

**(a)**

|  | **Main effects of Oxygen** | | **Main effects of Genotype** | | **Interaction effects between Oxygen and Genotype** | |
| --- | --- | --- | --- | --- | --- | --- |
| **SVZ** | F=5.670 | **p=0.009** | F=0.109 | p=0.744 | F=0.383 | p=0.686 |
| **VZ** | F=3.933 | **p=0.033** | F=0.329 | p=0.571 | F=2.603 | p=0.094 |

**(b)**

|  | | **Hypoxia** | **Physioxia** | **Hyperoxia** |
| --- | --- | --- | --- | --- |
| HIF-2α^flox/flox^ vs. HIF-2α CKO | **SVZ** | 0.730 | 0.395 | 0.950 |
|  | **VZ** | 0.342 | 0.935 | **0.027** |

|  | **SVZ** | | **VZ** | |
| --- | --- | --- | --- | --- |
|  | **HIF-2α^flox/flox^** | **HIF-2α CKO** | **HIF-2α^flox/flox^** | **HIF-2α CKO** |
| Hypoxia vs. Physioxia | 1.000 | 0.147 | **0.048** | 0.519 |
| Hypoxia vs. Hyperoxia | 0.111 | 0.054 | **0.021** | 1.000 |
| Physioxia vs. Hyperoxia | 0.660 | 1.000 | 1.000 | 0.255 |

**S6 Table. Statistics determined for phH3^+^/Tbr2^+^ cells per 200 µm column of E16.5 Emx1-Cre driven HIF-2α CKO mouse cortices exposed to different oxygen conditions (Fig 1h).** Cells were counted and analyzed separately for each zone (SVZ = Subventricular Zone, VZ = Ventricular Zone). **(a)** Main effects of oxygen and genotype, as well as interaction effects, on phH3^+^/Tbr2^+^ cells in the SVZ and the VZ were determined by two-way ANOVA. **(b)** Effects of genotype within each oxygen condition and of oxygen within each genotype were assessed using post-hoc *t*-tests. Displayed are Bonferroni-adjusted *p*-values. Bold values indicate significant differences.

**(a)**

|  | **Main effects of Oxygen** | | **Main effects of Genotype** | | **Interaction effects between Oxygen and Genotype** | |
| --- | --- | --- | --- | --- | --- | --- |
| **SVZ** | F=5.747 | **p=0.009** | F=0.176 | p=0.678 | F=0.149 | p=0.863 |
| **VZ** | F=0.312 | p=0.734 | F=1.766 | p=0.196 | F=0.692 | p=0.510 |

**(b)**

|  | | **Hypoxia** | **Physioxia** | **Hyperoxia** |
| --- | --- | --- | --- | --- |
| HIF-2α^flox/flox^ vs. HIF-2α CKO | **SVZ** | 0.887 | 0.473 | 0.926 |
|  | **VZ** | 0.212 | 0.348 | 0.904 |

|  | **SVZ** | | **VZ** | |
| --- | --- | --- | --- | --- |
|  | **HIF-2α^flox/flox^** | **HIF-2α CKO** | **HIF-2α^flox/flox^** | **HIF-2α CKO** |
| Hypoxia vs. Physioxia | 0.063 | 0.213 | 1.000 | 1.000 |
| Hypoxia vs. Hyperoxia | 0.162 | 0.123 | 1.000 | 1.000 |
| Physioxia vs. Hyperoxia | 1.000 | 1.000 | 1.000 | 0.747 |

**S7 Table. Statistics determined for Pax6^+^ cells per 200 µm column of E13.5 Emx1-Cre driven HIF-2α CKO mouse cortices exposed to different oxygen conditions (Fig 2c).** Cells were counted and analyzed separately for each zone (SVZ = Subventricular Zone, VZ = Ventricular Zone). **(a)** Main effects of oxygen and genotype, as well as interaction effects, on Pax6^+^ cells in the SVZ and the VZ were determined by two-way ANOVA or robust ANOVA (marked with *). **(b)** Effects of genotype within each oxygen condition and of oxygen within each genotype were assessed using post-hoc *t*-tests or Mann-Whitney U-tests. Displayed are Bonferroni-adjusted *p*-values. Bold values indicate significant differences.

**(a)**

|  | **Main effects of Oxygen** | | **Main effects of Genotype** | | **Interaction effects between Oxygen and Genotype** | |
| --- | --- | --- | --- | --- | --- | --- |
| **SVZ *** | F=2.646 | p=0.094 | F= 0.748 | p=0.397 | F=2.826 | p=0.082 |
| **VZ** | F=1.588 | p=0.228 | F=0.010 | p=0.923 | F=0.070 | p=0.932 |

**(b)**

|  | | **Hypoxia** | **Physioxia** | **Hyperoxia** |
| --- | --- | --- | --- | --- |
| HIF-2α^flox/flox^ vs. HIF-2α CKO | **SVZ *** | 0.413 | 0.905 | 0.286 |
|  | **VZ** | 0.789 | 0.891 | 0.812 |

|  | **SVZ *** | | **VZ** | |
| --- | --- | --- | --- | --- |
|  | **HIF-2α^flox/flox^** | **HIF-2α CKO** | **HIF-2α^flox/flox^** | **HIF-2α CKO** |
| Hypoxia vs. Physioxia | 1.000 | 0.570 | 1.000 | 1.000 |
| Hypoxia vs. Hyperoxia | 0.930 | 0.600 | 1.000 | 0.612 |
| Physioxia vs. Hyperoxia | 0.324 | 1.000 | 1.000 | 0.684 |

**S8 Table. Statistics determined for Tbr2^+^ cells per 200 µm column of E13.5 Emx1-Cre driven HIF-2α CKO mouse cortices exposed to different oxygen conditions (Fig  2d). Cells were counted and analyzed separately for each zone (SVZ = Subventricular Zone, VZ = Ventricular Zone).** **(a)** Main effects of oxygen and genotype, as well as interaction effects, on Tbr2^+^ cells in the SVZ and the VZ were determined by two-way ANOVA. **(b)** Effects of genotype within each oxygen condition and of oxygen within each genotype were assessed using post-hoc *t*-tests. Displayed are Bonferroni-adjusted *p*-values. Bold values indicate significant differences.

**(a)**

|  | **Main effects of Oxygen** | | **Main effects of Genotype** | | **Interaction effects between Oxygen and Genotype** | |
| --- | --- | --- | --- | --- | --- | --- |
| **SVZ** | F=0.765 | p=0.478 | F=0.149 | p=0.703 | F=1.929 | p=0.170 |
| **VZ** | F=0.012 | p=0.998 | F=0.142 | p=0.710 | F=0.193 | p=0.826 |

**(b)**

|  | | **Hypoxia** | **Physioxia** | **Hyperoxia** |
| --- | --- | --- | --- | --- |
| HIF-2α^flox/flox^ vs. HIF-2α CKO | **SVZ** | 0.245 | 0.129 | 0.778 |
|  | **VZ** | 0.480 | 0.927 | 0.980 |

|  | **SVZ** | | **VZ** | |
| --- | --- | --- | --- | --- |
|  | **HIF-2α^flox/flox^** | **HIF-2α CKO** | **HIF-2α^flox/flox^** | **HIF-2α CKO** |
| Hypoxia vs. Physioxia | 0.273 | 0.984 | 1.000 | 1.000 |
| Hypoxia vs. Hyperoxia | 1.000 | 0.771 | 1.000 | 1.000 |
| Physioxia vs. Hyperoxia | 0.432 | 1.000 | 1.000 | 1.000 |

**S9 Table. Statistics determined for phH3^+^ cells per 500 µm column of E13.5 Emx1-Cre driven HIF-2α CKO mouse cortices exposed to different oxygen conditions (Fig 2e).** Cells were counted and analyzed separately for each zone (SVZ = Subventricular Zone, VZ = Ventricular Zone). **(a)** Main effects of oxygen and genotype, as well as interaction effects, on phH3^+^ cells in the SVZ and the VZ were determined by two-way ANOVA. **(b)** Effects of genotype within each oxygen condition and of oxygen within each genotype were assessed using post-hoc *t*-tests. Displayed are Bonferroni-adjusted *p*-values. Bold values indicate significant differences.

**(a)**

|  | **Main effects of Oxygen** | | **Main effects of Genotype** | | **Interaction effects between Oxygen and Genotype** | |
| --- | --- | --- | --- | --- | --- | --- |
| **SVZ** | F=1.913 | p=0.172 | F=0.698 | p=0.413 | F=0.650 | p=0.532 |
| **VZ** | F=2.918 | p=0.076 | F=0.084 | p=0.775 | F=1.033 | p=0.373 |

**(b)**

|  | | **Hypoxia** | **Physioxia** | **Hyperoxia** |
| --- | --- | --- | --- | --- |
| HIF-2α^flox/flox^ vs. HIF-2α CKO | **SVZ** | 0.673 | 0.449 | 0.282 |
|  | **VZ** | 0.398 | 0.530 | 0.329 |

|  | **SVZ** | | **VZ** | |
| --- | --- | --- | --- | --- |
|  | **HIF-2α^flox/flox^** | **HIF-2α CKO** | **HIF-2α^flox/flox^** | **HIF-2α CKO** |
| Hypoxia vs. Physioxia | 0.309 | 1.000 | 1.000 | 1.000 |
| Hypoxia vs. Hyperoxia | 0.126 | 1.000 | 1.000 | 0.090 |
| Physioxia vs. Hyperoxia | 1.000 | 1.000 | 1.000 | 0.108 |

**S10 Table. Statistics determined for NeuN^+^ cells per 200 µm column of E16.5 Nestin-Cre driven HIF-2α CKO mouse cortices exposed to different oxygen conditions (Fig 3b).** Cells were counted and analyzed separately for each zone (SCP = Sub-Cortical Plate, DL = Deep Layers, UL = Upper Layers). **(a)** Main effects of oxygen and genotype, as well as interaction effects, on NeuN^+^ cells in SCP, DL, and UL were determined by two-way ANOVA. **(b)** Effects of genotype within each oxygen condition and of oxygen within each genotype were assessed using post-hoc *t*-tests. Displayed are Bonferroni-adjusted *p*-values. Bold values indicate significant differences.

**(a)**

|  | **Main effects of Oxygen** | | **Main effects of Genotype** | | **Interaction effects between Oxygen and Genotype** | |
| --- | --- | --- | --- | --- | --- | --- |
| **SCP** | F=3.669 | p=0.073 | F=1.251 | p=0.280 | F=1.308 | p=0.270 |
| **DL** | F=4.304 | p=0.055 | F=0.185 | p=0.673 | F=0.028 | p=0.870 |
| **UL** | F=2.603 | p=0.126 | F=0.146 | p=0.708 | F=0.076 | p=0.786 |

**(b)**

|  | | **Hypoxia** | **Physioxia** |
| --- | --- | --- | --- |
| HIF-2α^flox/flox^ vs. HIF-2α CKO | **SCP** | 0.986 | 0.129 |
|  | **DL** | 0.679 | 0.854 |
|  | **UL** | 0.941 | 0.648 |

|  | | **HIF-2α^flox/flox^** | **HIF-2α CKO** |
| --- | --- | --- | --- |
| Hypoxia vs. Physioxia | **SCP** | 0.593 | **0.046** |
|  | **DL** | 0.133 | 0.196 |
|  | **UL** | 0.358 | 0.200 |

**S11 Table. Statistics determined for BrdU^+^/NeuN^+^ cells per 200 µm column of E16.5 Nestin-Cre driven HIF-2α CKO mouse cortices exposed to different oxygen conditions (Fig 3c).** Cells were counted and analyzed separately for each zone (SCP = Sub-Cortical Plate, DL = Deep Layers, UL = Upper Layers). **(a)** Main effects of oxygen and genotype, as well as interaction effects, on BrdU^+^/NeuN^+^ cells in SCP, DL, and UL were determined by two-way ANOVA. **(b)** Effects of genotype within each oxygen condition and of oxygen within each genotype were assessed using post-hoc *t*-tests. Displayed are Bonferroni-adjusted *p*-values. Bold values indicate significant differences.

**(a)**

|  | **Main effects of Oxygen** | | **Main effects of Genotype** | | **Interaction effects between Oxygen and Genotype** | |
| --- | --- | --- | --- | --- | --- | --- |
| **SCP** | F=0.000 | p=0.984 | F=0.127 | p=0.727 | F=0.001 | p=0.978 |
| **DL** | F=24.857 | **p<0.001** | F=3.483 | p=0.087 | F=3.435 | p=0.089 |
| **UL** | F=2.316 | p=0.148 | F=0.002 | p=0.963 | F=1.126 | p=0.304 |

**(b)**

|  | | **Hypoxia** | **Physioxia** |
| --- | --- | --- | --- |
| HIF-2α^flox/flox^ vs. HIF-2α CKO | **SCP** | 0.820 | 0.790 |
|  | **DL** | 0.993 | **0.022** |
|  | **UL** | 0.445 | 0.749 |

|  | | **HIF-2α^flox/flox^** | **HIF-2α CKO** |
| --- | --- | --- | --- |
| Hypoxia vs. Physioxia | **SCP** | 0.996 | 0.973 |
|  | **DL** | **<0.001** | **0.047** |
|  | **UL** | 0.087 | 0.749 |

**S12 Table. Statistics determined for Tbr1^+^ cells per 200 µm column of E16.5 Emx1-Cre driven HIF-2α CKO mouse cortices exposed to different oxygen conditions (Fig 4b).** **(a)** Main effects of oxygen and genotype, as well as the interaction effect, on Tbr1^+^ cells were determined by two-way ANOVA. **(b)** Effects of genotype within each oxygen condition and of oxygen within each genotype were assessed using post-hoc *t*-tests. Displayed are Bonferroni-adjusted *p*-values. Bold values indicate significant differences.

**(a)**

| **Main effect of Oxygen** | | **Main effect of Genotype** | | **Interaction effect between Oxygen and Genotype** | |
| --- | --- | --- | --- | --- | --- |
| F=4.921 | **p=0.018** | F=1.102 | p=0.306 | F=0.696 | p=0.510 |

**(b)**

|  | **Hypoxia** | **Physioxia** | **Hyperoxia** |
| --- | --- | --- | --- |
| HIF-2α^flox/flox^ vs. HIF-2α CKO | 0.957 | 0.861 | 0.146 |

|  | **HIF-2α^flox/flox^** | **HIF-2α CKO** |
| --- | --- | --- |
| Hypoxia vs. Physioxia | 1.000 | 1.000 |
| Hypoxia vs. Hyperoxia | **0.024** | 0.543 |
| Physioxia vs. Hyperoxia | 0.108 | 1.000 |

**S13 Table. Statistics determined for Ctip2^+^/Tbr1^-^ cells per 200 µm column of E16.5 Emx1-Cre driven HIF-2α CKO mouse cortices exposed to different oxygen conditions (Fig 4c).** **(a)** Main effects of oxygen and genotype, as well as the interaction effect, on Ctip2^+^/Tbr1^-^ cells were determined by two-way ANOVA. **(b)** Effects of genotype within each oxygen condition and of oxygen within each genotype were assessed using post-hoc *t*-tests. Displayed are Bonferroni-adjusted *p*-values. Bold values indicate significant differences.

**(a)**

| **Main effect of Oxygen** | | **Main effect of Genotype** | | **Interaction effect between Oxygen and Genotype** | |
| --- | --- | --- | --- | --- | --- |
| F=10.016 | **p<0.001** | F=0.008 | p=0.930 | F=0.839 | p=0.446 |

**(b)**

|  | **Hypoxia** | **Physioxia** | **Hyperoxia** |
| --- | --- | --- | --- |
| HIF-2α^flox/flox^ vs. HIF-2α CKO | 0.957 | 0.384 | 0.357 |

|  | **HIF-2α^flox/flox^** | **HIF-2α CKO** |
| --- | --- | --- |
| Hypoxia vs. Physioxia | 1.000 | 0.222 |
| Hypoxia vs. Hyperoxia | **0.006** | **0.042** |
| Physioxia vs. Hyperoxia | **0.045** | 1.000 |

**S14 Table. Statistics determined for Satb2^+^ cells per 200 µm column of E16.5 Emx1-Cre driven HIF-2α CKO mouse cortices exposed to different oxygen conditions (Fig 4d).** **(a)** Main effects of oxygen and genotype, as well as the interaction effect, on Satb2^+^ cells were determined by two-way ANOVA. **(b)** Effects of genotype within each oxygen condition and of oxygen within each genotype were assessed using post-hoc *t*-tests. Displayed are Bonferroni-adjusted *p*-values. Bold values indicate significant differences.

**(a)**

| **Main effect of Oxygen** | | **Main effect of Genotype** | | **Interaction effect between Oxygen and Genotype** | |
| --- | --- | --- | --- | --- | --- |
| F=8.885 | **p=0.002** | F=2.979 | p=0.099 | F=3.068 | p=0.068 |

**(b)**

|  | **Hypoxia** | **Physioxia** | **Hyperoxia** |
| --- | --- | --- | --- |
| HIF-2α^flox/flox^ vs. HIF-2α CKO | 0.217 | 0.385 | **0.019** |

|  | **HIF-2α^flox/flox^** | **HIF-2α CKO** |
| --- | --- | --- |
| Hypoxia vs. Physioxia | 1.000 | 0.318 |
| Hypoxia vs. Hyperoxia | **0.006** | 0.138 |
| Physioxia vs. Hyperoxia | **0.003** | 1.000 |

**S15 Table. Statistics determined for NSC counts *in vitro* derived from E14.5 NesCr-HIF-2α CKO and respective control mouse cortices cultures under physioxia (3% pO_2_) and hyperoxia (21% pO_2_) for up to 6 days (Fig 5b).** The cell counts on days 4 and 6 were analyzed separately. **(a)** Main effects of oxygen and genotype, as well as interaction effects, were determined by robust two-way ANOVA. **(b)** Effects of genotype within each oxygen condition and of oxygen within each genotype were assessed using post-hoc Mann-Whitney U-tests (marked with *). Displayed are Bonferroni-adjusted *p*-values. Bold values indicate significant differences.

**(a)**

|  | **Main effects of Oxygen** | | **Main effects of Genotype** | | **Interaction effects between Oxygen and Genotype** | |
| --- | --- | --- | --- | --- | --- | --- |
| **d4** | F=3.498 | p=0.068 | F= 0.005 | p=0.942 | F=0.411 | p=0.525 |
| **d6** | F=34.919 | **p<0.001** | F= 0.040 | p=0.841 | F=1.452 | p=0.234 |

**(b)**

|  | | **Physioxia** | **Hyperoxia** | |
| --- | --- | --- | --- | --- |
| HIF-2α^flox/flox^ vs. HIF-2α CKO | **d4** | 0.661 | 0.446 |  |
|  | **d6** | 0.501 | 0.262 |  |

|  | | **HIF-2α^flox/flox^** | **HIF-2α CKO** | |  |
| --- | --- | --- | --- | --- | --- |
| Physioxia vs. Hyperoxia | **d4** | **0.018** | | 0.508 | |
|  | **d6** | **0.001** | | **0.023** | |

**S16 Table. Statistics determined for Nestin^+^ and β-Tubulin^+^ cells (% of total cells) and gene expression of *Olig2* and *Map2* in NSC cultures *in vitro* derived from E14.5 NesCr-HIF-2α CKO and respective control mouse cortices cultures under physioxia (3% pO_2_) and hyperoxia (21% pO_2_; Fig 6c).** **(a)** Main effects of oxygen and genotype, as well as interaction effects, on Nestin^+^ and β-Tubulin^+^ cells and gene expression of *Olig2* and *Map2* were determined by two-way ANOVA. **(b)** Effects of genotype within each oxygen condition and of oxygen within each genotype were assessed using post-hoc *t*-tests. Displayed are Bonferroni-adjusted *p*-values. Bold values indicate significant differences.

**(a)**

|  | **Main effects of Oxygen** | | **Main effects of Genotype** | | **Interaction effects between Oxygen and Genotype** | |
| --- | --- | --- | --- | --- | --- | --- |
| β-Tubulin^+^-NSCs | F=17.211 | **p<0.001** | F= 1.724 | p=0.201 | F=1.720 | p=0.202 |
| Nestin^+^-NSCs | F=15.618 | **p=0.001** | F= 2.449 | p=0.130 | F=0.103 | p=0.751 |
| Olig2 | F=1.290 | p=0.272 | F= 0.932 | p=0.348 | F=0.185 | p=0.672 |
| Map2 | F=0.005 | p=0.947 | F= 0.273 | p=0.609 | F=1.432 | p=0.250 |

**(b)**

|  | | **Physioxia** | **Hyperoxia** |
| --- | --- | --- | --- |
| HIF-2α^flox/flox^ vs. HIF-2α CKO | β-Tubulin^+^-NSCs | 0.080 | 0.999 |
|  | Nestin^+^-NSCs | 0.203 | 0.377 |
|  | Olig2 | 0.327 | 0.716 |
|  | Map2 | 0.638 | 0.247 |

|  | | **HIF-2α^flox/flox^** | **HIF-2α CKO** |
| --- | --- | --- | --- |
| Physioxia vs. Hyperoxia | β-Tubulin^+^-NSCs | 0.052 | **0.001** |
|  | Nestin^+^-NSCs | **0.019** | 0.377 |
|  | Olig2 | 0.617 | 0.294 |
|  | Map2 | 0.408 | 0.413 |

**S17 Table. Statistics determined for gene expression of *Tcf1*, *Lef1*, *Notch1,* and *Hes1* in NSC cultures *in vitro* derived from E14.5 NesCr-HIF-2α CKO and respective control mouse cortices cultures under physioxia (3% pO_2_) and hyperoxia (21% pO_2_; Fig 6d).** **(a)** Main effects of oxygen and genotype, as well as interaction effects, on gene expression of *Tcf1*, *Lef1*, *Notch1,* and *Hes1* were determined by two-way ANOVA or robust two-way ANOVA (marked with *). **(b)** Effects of genotype within each oxygen condition and of oxygen within each genotype were assessed using post-hoc *t*-tests or Mann-Whitney U-tests (marked with *). Displayed are Bonferroni-adjusted *p*-values. Bold values indicate significant differences.

**(a)**

|  | **Main effects of Oxygen** | | **Main effects of Genotype** | | **Interaction effects between Oxygen and Genotype** | |
| --- | --- | --- | --- | --- | --- | --- |
| Tcf1 | F=0.001 | p=0.976 | F=1.686 | p=0.209 | F=0.471 | p=0.501 |
| Lef1 | F=0.305 | p=0.587 | F=1.154 | p=0.296 | F=0.488 | p=0.493 |
| Notch1* | F=1.855 | p=0.181 | F=0.007 | p=0.933 | F=0.310 | p=0.581 |
| Hes1 | F=0.701 | p=0.415 | F=0.248 | p=0.626 | F=4.162 | **p=0.047** |

**(b)**

|  | | **Physioxia** | **Hyperoxia** |
| --- | --- | --- | --- |
| HIF-2α^flox/flox^ vs. HIF-2α CKO | Tcf1 | 0.670 | 0.176 |
|  | Lef1 | 0.788 | 0.236 |
|  | Notch1* | 1 | 0.537 |
|  | Hes1 | 0.256 | 0.083 |

|  | | **HIF-2α^flox/flox^** | **HIF-2α CKO** |
| --- | --- | --- | --- |
| Physioxia vs. Hyperoxia | Tcf1 | 0.648 | 0.618 |
|  | Lef1 | 0.398 | 0.917 |
|  | Notch1* | 0.556 | 0.818 |
|  | Hes1 | 0.062 | 0.343 |

**S18 Table. Statistics determined for *HIF-2α* expression on the DNA- and mRNA level *in vivo* of E16.5 Nestin-Cre driven HIF-2α CKO mouse cortices exposed to physioxia (21% pO_2_; S1 Fig a).** **(a)** Effects of genotype on *HIF‑2α* DNA- and mRNA levels were determined by a *t*-test or a Mann-Whitney U-test (marked with *).

**(a)**

| **Expression of HIF-2α *in vivo*** | HIF-2α^flox/flox^ vs. HIF-2α CKO |
| --- | --- |
| DNA * | **p<0.001** |
| RNA | **p<0.001** |

**S19 Table. Statistics determined for *Slc2a1*, *Vegf,* and *HIF-1α* expression on the mRNA level *in vivo* of E16.5 Nestin-Cre driven HIF-2α CKO mouse cortices exposed to physioxia (21% pO_2_; S1 Fig b).** **(a)** Effects of genotype on mRNA levels were determined by *t*-tests.

**(a)**

| **Expression *in vivo*** | HIF-2α^flox/flox^ vs. HIF-2α CKO |
| --- | --- |
| *Slc2a1* | p=0.839 |
| *Vegf* | p=0.206 |
| *HIF-1α* | p=0.780 |

**S20 Table. Statistics determined for *Sox2*, *Lef1,* and *Tcf1* expression on the mRNA level *in vivo* of E16.5 Nestin-Cre driven HIF-2α CKO mouse cortices exposed to physioxia (21% pO_2_; S1 Fig c).** **(a)** Effects of genotype on mRNA levels were determined by *t*-tests.

**(a)**

| **Expression *in vivo*** | HIF-2α^flox/flox^ vs. HIF-2α CKO |
| --- | --- |
| *Sox2* | p=0.844 |
| *Lef1* | p=0.525 |
| *Tcf1* | p=0.808 |

**S21 Table. Statistics determined for expression of HIF-2α-Protein, -RNA and HIF-1α mRNA level in NSC cultures *in vitro* derived from E14.5 NesCr-HIF-2α CKO and respective control mouse cortices cultures under physioxia (3% pO_2_, green) and hyperoxia (21% pO_2_, red; S2 Fig a). (a)** Main effects of oxygen and genotype, as well as interaction effects, were determined by two-way ANOVA or by robust two-way ANOVA (marked with *). **(b)** Effects of genotype within each oxygen condition and of oxygen within each genotype were assessed using post-hoc *t*-tests. Displayed are Bonferroni-adjusted *p*-values. Bold values indicate significant differences.

**(a)**

|  | **Main effects of Oxygen** | | **Main effects of Genotype** | | **Interaction effects between Oxygen and Genotype** | |
| --- | --- | --- | --- | --- | --- | --- |
| HIF-2α-Protein* | F=0.0167 | p=0.899 | F=8.486 | **p=0.007** | F=0.684 | p=0.416 |
| HIF-2α-RNA* | F=38.253 | **p<0.001** | F=120.734 | **p<0.001** | F=38.378 | **p<0.001** |
| HIF-1α-RNA | F=0.136 | p=0.717 | F=0.155 | p=0.699 | F=0.714 | p=0.410 |

**(b)**

|  | | **Physioxia** | **Hyperoxia** |
| --- | --- | --- | --- |
| HIF-2α^flox/flox^ vs. HIF-2α CKO | HIF-2α-Protein* | **0.002** | 0.088 |
|  | HIF-2α-RNA* | **0.002** | **0.001** |
|  | HIF-1α-RNA | 0.383 | 0.758 |

**S22 Table.** Statistics determined for gene expression of *Slc2a1*, *Vegf,* and *Sox2* in NSC cultures *in vitro* derived from E14.5 NesCr-HIF-2α CKO and respective control mouse cortices cultures under physioxia (3% pO_2_, green) and hyperoxia (21% pO_2_, red; **S2 Fig b).** **(a)** Main effects of oxygen and genotype, as well as interaction effects, were determined by two-way ANOVA. **(b)** Effects of genotype within each oxygen condition and of oxygen within each genotype were assessed using post-hoc *t*-tests. Displayed are Bonferroni-adjusted *p*-values. Bold values indicate significant differences

**(a)**

|  | **Main effects of Oxygen** | | **Main effects of Genotype** | | **Interaction effects between Oxygen and Genotype** | |
| --- | --- | --- | --- | --- | --- | --- |
| Slc2a1 | F=45.758 | **p<0.001** | F=5.067 | **p=0.036** | F=3.334 | p=0.084 |
| Vegf | F=45.048 | **p<0.001** | F=5.802 | **p=0.026** | F=0.842 | p=0.370 |
| Sox2 | F=2.405 | p=0.140 | F=1.562 | p=0.229 | F=0.133 | p=0.720 |

**(b)**

|  | | **Physioxia** | **Hyperoxia** |
| --- | --- | --- | --- |
| HIF-2α^flox/flox^ vs. HIF-2α CKO | Slc2a1 | **0.008** | 0.772 |
|  | Vegf | **0.026** | 0.554 |
|  | Sox2 | 0.246 | 0.559 |

|  | | **HIF-2α^flox/flox^** | **HIF-2α CKO** |
| --- | --- | --- | --- |
| Physioxia vs. Hyperoxia | Slc2a1 | **<0.001** | **0.002** |
|  | Vegf | **<0.001** | **0.001** |
|  | Sox2 | 0.409 | 0.199 |

**S23 Table. Statistics determined for Pax6^+^ cells per 200 µm column of E16.5 NesCr-HIF-2α mouse cortices exposed to different oxygen conditions (S3 Fig a).** Cells were counted and analyzed separately for each zone (SVZ = Subventricular Zone, VZ = Ventricular Zone). **(a)** Main effects of oxygen and genotype, as well as interaction effects, on Pax6^+^ cells in the SVZ and the VZ were determined by two-way ANOVA. **(b)** Effects of genotype within each oxygen condition and of oxygen within each genotype were assessed using post-hoc *t*-tests. Displayed are Bonferroni-adjusted *p*-values. Bold values indicate significant differences.

**(a)**

|  | **Main effects of Oxygen** | | **Main effects of Genotype** | | **Interaction effects between Oxygen and Genotype** | |
| --- | --- | --- | --- | --- | --- | --- |
| **SVZ** | F=0.001 | p=0.974 | F=0.014 | p=0.907 | F=0.834 | p=0.381 |
| **VZ** | F=5.781 | **p=0.035** | F=2.788 | p=0.123 | F=1.064 | p=0.324 |

**(b)**

|  | | **Hypoxia** | **Physioxia** |
| --- | --- | --- | --- |
| HIF-2α^flox/flox^ vs. HIF-2α CKO | **SVZ** | 0.599 | 0.463 |
|  | **VZ** | 0.093 | 0.648 |

|  | | **HIF-2α^flox/flox^** | **HIF-2α CKO** |
| --- | --- | --- | --- |
| Hypoxia vs. Physioxia | **SVZ** | 0.532 | 0.530 |
|  | **VZ** | **0.039** | 0.334 |

**S24 Table. Statistics determined for Tbr2^+^ cells per 200 µm column of E16.5 NesCr-HIF-2α mouse cortices exposed to different oxygen conditions (S3 Fig b).** Cells were counted and analyzed separately for each zone (SVZ = Subventricular Zone, VZ = Ventricular Zone). **(a)** Main effects of oxygen and genotype, as well as interaction effects, on Tbr2^+^ cells in the SVZ and the VZ were determined by two-way ANOVA or robust two-way ANOVA (marked with *). **(b)** Effects of genotype within each oxygen condition and of oxygen within each genotype were assessed using post-hoc *t*-tests. Displayed are Bonferroni-adjusted *p*-values. Bold values indicate significant differences.

**(a)**

|  | **Main effects of Oxygen** | | **Main effects of Genotype** | | **Interaction effects between Oxygen and Genotype** | |
| --- | --- | --- | --- | --- | --- | --- |
| **SVZ** | F=4.960 | **p=0.044** | F=0.370 | p=0.850 | F=0.000 | p=0.983 |
| **VZ *** | F=6.370 | **p=0.025** | F=0.730 | p=0.408 | F=0.000 | p=0.999 |

**(b)**

|  | | **Hypoxia** | **Physioxia** |
| --- | --- | --- | --- |
| HIF-2α^flox/flox^ vs. HIF-2α CKO | **SVZ** | 0.886 | 0.901 |
|  | **VZ *** | 0.549 | 0.902 |

|  | | **HIF-2α^flox/flox^** | **HIF-2α CKO** |
| --- | --- | --- | --- |
| Hypoxia vs. Physioxia | **SVZ** | 0.168 | 0.108 |
|  | **VZ *** | 0.095 | 0.176 |

**S25 Table. Statistics determined for phH3^+^ cells per 500 µm column of E16.5 NesCr-HIF-2α mouse cortices exposed to different oxygen conditions (S3 Fig c).** Cells were counted and analyzed separately for each zone (SVZ = Subventricular Zone, VZ = Ventricular Zone). **(a)** Main effects of oxygen and genotype, as well as interaction effects, on phH3^+^ cells in the SVZ and the VZ were determined by two-way ANOVA. **(b)** Effects of genotype within each oxygen condition and of oxygen within each genotype were assessed using post-hoc *t*-tests. Displayed are Bonferroni-adjusted *p*-values. Bold values indicate significant differences.

**(a)**

|  | **Main effects of Oxygen** | | **Main effects of Genotype** | | **Interaction effects between Oxygen and Genotype** | |
| --- | --- | --- | --- | --- | --- | --- |
| **SVZ** | F=2.992 | p=0.107 | F=1.003 | p=0.335 | F=0.427 | p=0.525 |
| **VZ** | F=0.923 | p=0.354 | F=0.027 | p=0.871 | F=0.001 | p=0.974 |

**(b)**

|  | | **Hypoxia** | **Physioxia** |
| --- | --- | --- | --- |
| HIF-2α^flox/flox^ vs. HIF-2α CKO | **SVZ** | 0.281 | 0.801 |
|  | **VZ** | 0.930 | 0.885 |

|  | | **HIF-2α^flox/flox^** | **HIF-2α CKO** |
| --- | --- | --- | --- |
| Hypoxia vs. Physioxia | **SVZ** | 0.497 | 0.084 |
|  | **VZ** | 0.557 | 0.450 |

**S26 Table. Statistics determined for Pax6^+^ cells per 200 µm column of E16.5 Emx1-Cre driven HIF-2α CKO mouse retrosplenial cortices exposed to different oxygen conditions (S4 Fig b).** Cells were counted and analyzed separately for each zone (SVZ = Subventricular Zone, VZ = Ventricular Zone). **(a)** Main effects of oxygen and genotype, as well as interaction effects, on Pax6^+^ cells in the SVZ and the VZ were determined by two-way ANOVA or robust two-way ANOVA (marked with *). **(b)** Effects of genotype within each oxygen condition and of oxygen within each genotype were assessed using post-hoc *t*-tests. Displayed are Bonferroni-adjusted *p*-values. Bold values indicate significant differences.

**(a)**

|  | **Main effects of Oxygen** | | **Main effects of Genotype** | | **Interaction effects between Oxygen and Genotype** | |
| --- | --- | --- | --- | --- | --- | --- |
| **SVZ*** | F=0.341 | p=0.714 | F=0.198 | p=0.660 | F=1.253 | p=0.301 |
| **VZ** | F=0.507 | p=0.608 | F=1.650 | p=0.210 | F=0.549 | p=0.584 |

**(b)**

|  | | **Hypoxia** | **Physioxia** | **Hyperoxia** |
| --- | --- | --- | --- | --- |
| HIF-2α^flox/flox^ vs. HIF-2α CKO | **SVZ *** | 0.111 | 0.429 | 0.282 |
|  | **VZ** | 0.907 | 0.122 | 0.573 |

|  | | **HIF-2α^flox/flox^** | **HIF-2α CKO** |
| --- | --- | --- | --- |
| Hypoxia vs. Physioxia | **SVZ *** | 1.000 | 1.000 |
|  | **VZ** | 0.969 | 1.000 |
| Hypoxia vs. Hyperoxia | **SVZ *** | 1.000 | 1.000 |
|  | **VZ** | 1.000 | 1.000 |
| Physioxia vs. Hyperoxia | **SVZ *** | 1.000 | 0.531 |
|  | **VZ** | 1.000 | 1.000 |

**S27 Table. Statistics determined for Tbr2^+^ cells per 200 µm column of E16.5 Emx1-Cre driven HIF-2α CKO mouse retrosplenial cortices exposed to different oxygen conditions (S4 Fig c).** Cells were counted and analyzed separately for each zone (SVZ = Subventricular Zone, VZ = Ventricular Zone). **(a)** Main effects of oxygen and genotype, as well as interaction effects, on Tbr2^+^ cells in the SVZ and the VZ were determined by two-way ANOVA. **(b)** Effects of genotype within each oxygen condition and of oxygen within each genotype were assessed using post-hoc *t*-tests. Displayed are Bonferroni-adjusted *p*-values. Bold values indicate significant differences.

**(a)**

|  | **Main effects of Oxygen** | | **Main effects of Genotype** | | **Interaction effects between Oxygen and Genotype** | |
| --- | --- | --- | --- | --- | --- | --- |
| **SVZ** | F=0.348 | p=0.709 | F=0.461 | p=0.503 | F=0.142 | p=0.868 |
| **VZ** | F=0.563 | p=0.576 | F=0.072 | p=0.791 | F=0.082 | p=0.922 |

**(b)**

|  | | **Hypoxia** | **Physioxia** | **Hyperoxia** |
| --- | --- | --- | --- | --- |
| HIF-2α^flox/flox^ vs. HIF-2α CKO | **SVZ** | 0.990 | 0.694 | 0.397 |
|  | **VZ** | 0.939 | 0.928 | 0.610 |

|  | | **HIF-2α^flox/flox^** | **HIF-2α CKO** |
| --- | --- | --- | --- |
| Hypoxia vs. Physioxia | **SVZ** | 1.000 | 1.000 |
|  | **VZ** | 1.000 | 1.000 |
| Hypoxia vs. Hyperoxia | **SVZ** | 1.000 | 1.000 |
|  | **VZ** | 1.000 | 1.000 |
| Physioxia vs. Hyperoxia | **SVZ** | 1.000 | 1.000 |
|  | **VZ** | 1.000 | 1.000 |

**S28 Table. Statistics determined for Pax6^+^/Tbr2^+^ cells per 200 µm column of E16.5 Emx1-Cre driven HIF-2α CKO mouse retrosplenial cortices exposed to different oxygen conditions (S4 Fig d).** Cells were counted and analyzed separately for each zone (SVZ = Subventricular Zone, VZ = Ventricular Zone). **(a)** Main effects of oxygen and genotype, as well as interaction effects, on Pax6^+^/Tbr2^+^ cells in the SVZ and the VZ were determined by two-way ANOVA. **(b)** Effects of genotype within each oxygen condition and of oxygen within each genotype were assessed using post-hoc *t*-tests. Displayed are Bonferroni-adjusted *p*-values. Bold values indicate significant differences.

**(a)**

|  | **Main effects of Oxygen** | | **Main effects of Genotype** | | **Interaction effects between Oxygen and Genotype** | |
| --- | --- | --- | --- | --- | --- | --- |
| **SVZ** | F=0.716 | p=0.498 | F=0.362 | p=0.552 | F=0.165 | p=0.849 |
| **VZ** | F=0.851 | p=0.438 | F=0.453 | p=0.507 | F=0.263 | p=0.771 |

**(b)**

|  | | **Hypoxia** | **Physioxia** | **Hyperoxia** |
| --- | --- | --- | --- | --- |
| HIF-2α^flox/flox^ vs. HIF-2α CKO | **SVZ** | 0.481 | 0.996 | 0.815 |
|  | **VZ** | 0.704 | 0.912 | 0.344 |

|  | | **HIF-2α^flox/flox^** | **HIF-2α CKO** |
| --- | --- | --- | --- |
| Hypoxia vs. Physioxia | **SVZ** | 1.000 | 0.870 |
|  | **VZ** | 1.000 | 1.000 |
| Hypoxia vs. Hyperoxia | **SVZ** | 1.000 | 1.000 |
|  | **VZ** | 1.000 | 1.000 |
| Physioxia vs. Hyperoxia | **SVZ** | 1.000 | 1.000 |
|  | **VZ** | 1.000 | 0.558 |

**S29 Table. Statistics determined for phH3^+^ cells per 500 µm column of E16.5 Emx1-Cre driven HIF-2α CKO mouse retrosplenial cortices exposed to different oxygen conditions (S4 Fig e).** Cells were counted and analyzed separately for each zone (SVZ = Subventricular Zone, VZ = Ventricular Zone). **(a)** Main effects of oxygen and genotype, as well as interaction effects, on phH3^+^ cells in the SVZ and the VZ were determined by two-way ANOVA. **(b)** Effects of genotype within each oxygen condition and of oxygen within each genotype were assessed using post-hoc *t*-tests. Displayed are Bonferroni-adjusted *p*-values. Bold values indicate significant differences.

**(a)**

|  | **Main effects of Oxygen** | | **Main effects of Genotype** | | **Interaction effects between Oxygen and Genotype** | |
| --- | --- | --- | --- | --- | --- | --- |
| **SVZ** | F=0.142 | p=0.868 | F=0.671 | p=0.420 | F=0.001 | p=0.999 |
| **VZ** | F=2.511 | p=0.102 | F=0.231 | p=0.635 | F=1.067 | p=0.360 |

**(b)**

|  | | **Hypoxia** | **Physioxia** | **Hyperoxia** |
| --- | --- | --- | --- | --- |
| HIF-2α^flox/flox^ vs. HIF-2α CKO | **SVZ** | 0.678 | 0.653 | 0.576 |
|  | **VZ** | 0.473 | 0.378 | 0.312 |

|  | | **HIF-2α^flox/flox^** | **HIF-2α CKO** |
| --- | --- | --- | --- |
| Hypoxia vs. Physioxia | **SVZ** | 1.000 | 1.000 |
|  | **VZ** | 1.000 | 1.000 |
| Hypoxia vs. Hyperoxia | **SVZ** | 1.000 | 1.000 |
|  | **VZ** | 0.471 | 0.690 |
| Physioxia vs. Hyperoxia | **SVZ** | 1.000 | 1.000 |
|  | **VZ** | 0.054 | 1.000 |

**S30 Table. Statistics determined for vessel density within the cortical area of E16.5 Nestin-Cre driven HIF-2α CKO mouse cortices exposed to different oxygen conditions (S5 Fig c).** Vessel density was assessed in the whole cortex. **(a)** Main effects of oxygen and genotype, as well as interaction effects, on vessel density were determined by two-way ANOVA. **(b)** Effects of genotype within each oxygen condition and of oxygen within each genotype were assessed using post-hoc *t*-tests. Displayed are Bonferroni-adjusted *p*-values. Bold values indicate significant differences.

**(a)**

| **Main effects of Oxygen** | | **Main effects of Genotype** | | **Interaction effects between Oxygen and Genotype** | |
| --- | --- | --- | --- | --- | --- |
| F=2.027 | p=0.182 | F=0.005 | p=0.944 | F=3.818 | p=0.077 |

**(b)**

|  | **Hypoxia** | **Physioxia** |
| --- | --- | --- |
| HIF-2α^flox/flox^ vs. HIF-2α CKO | 0.195 | 0.193 |

|  | **HIF-2α^flox/flox^** | **HIF-2α CKO** |
| --- | --- | --- |
| Hypoxia vs. Physioxia | 0.725 | **0.030** |

**S31 Table. Statistics determined for apoptotic CC3^+^ cells within the cortical area of E16.5 Nestin-Cre driven HIF-2α CKO mouse cortices exposed to different oxygen conditions (S5 Fig d).** The number of apoptotic CC3^+^ cells was assessed in the whole cortex. **(a)** Main effects of oxygen and genotype, as well as interaction effects, on CC3^+^ cells were determined by two-way ANOVA. **(b)** Effects of genotype within each oxygen condition and of oxygen within each genotype were assessed using post-hoc *t*-tests. Displayed are Bonferroni-adjusted *p*-values. Bold values indicate significant differences.

**(a)**

|  | **Main effects of Oxygen** | | **Main effects of Genotype** | | **Interaction effects between Oxygen and Genotype** | |
| --- | --- | --- | --- | --- | --- | --- |
| **Cortical Plate** | F=0.659 | p=0.433 | F=2.216 | p=0.162 | F=0.051 | p=0.825 |
| **Apical** | F=6.809 | **p=0.023** | F=0.019 | p=0.894 | F=0.034 | p=0.856 |

**(b)**

|  | | **Hypoxia** | **Physioxia** |
| --- | --- | --- | --- |
| HIF-2α^flox/flox^ vs. HIF-2α CKO | **Cortical Plate** | 0.389 | 0.249 |
|  | **Apical** | 0.973 | 0.824 |

|  | | **HIF-2α^flox/flox^** | **HIF-2α CKO** |
| --- | --- | --- | --- |
| Hypoxia vs. Physioxia | **Cortical Plate** | 0.477 | 0.686 |
|  | **Apical** | 0.072 | 0.112 |

**S32 Table. Statistics determined for NeuN^+^ cells per 200 µm column of E16.5 Emx1-Cre driven HIF-2α CKO mouse cortices exposed to different oxygen conditions (S6 Fig b).** Cells were counted and analyzed separately for each zone (SCP = Sub-Cortical Plate, DL = Deep Layers, UL = Upper Layers). **(a)** Main effects of oxygen and genotype, as well as interaction effects, on NeuN^+^ cells in SCP, DL, and UL were determined by two-way ANOVA or robust two-way ANOVA (marked with *). **(b)** Effects of genotype within each oxygen condition and of oxygen within each genotype were assessed using post-hoc *t*-tests or Mann-Whitney U-test (marked with *). Displayed are Bonferroni-adjusted *p*-values. Bold values indicate significant differences.

**(a)**

|  | **Main effects of Oxygen** | | **Main effects of Genotype** | | **Interaction effects between Oxygen and Genotype** | |
| --- | --- | --- | --- | --- | --- | --- |
| **SCP** | F=0.516 | p=0.604 | F=2.427 | p=0.134 | F=3.714 | **p=0.042** |
| **DL** | F=1.476 | p=0.251 | F=0.205 | p=0.655 | F=1.054 | p=0.366 |
| **UL *** | F=1.855 | p=0.181 | F=0.007 | p=0.933 | F=0.310 | p=0.581 |

**(b)**

|  | | **Hypoxia** | **Physioxia** | **Hyperoxia** |
| --- | --- | --- | --- | --- |
| HIF-2α^flox/flox^ vs. HIF-2α CKO | **SCP** | 0.242 | 0.252 | **0.017** |
|  | **DL** | 0.783 | 0.437 | 0.226 |
|  | **UL *** | 0.343 | 0.413 | 0.413 |

|  | | **HIF-2α^flox/flox^** | **HIF-2α CKO** |
| --- | --- | --- | --- |
| Hypoxia vs. Physioxia | **SCP** | 0.234 | 1.000 |
|  | **DL** | 1.000 | 0.309 |
|  | **UL *** | 0.086 | 1.000 |
| Hypoxia vs. Hyperoxia | **SCP** | 1.000 | 0.765 |
|  | **DL** | 0.819 | 1.000 |
|  | **UL *** | 0.086 | 1.000 |
| Physioxia vs. Hyperoxia | **SCP** | 0.123 | 0.354 |
|  | **DL** | 1.000 | 0.408 |
|  | **UL *** | 1.000 | 1.000 |
